# *Salmonella enterica* subsp. II serovar 4,5,12:a:- may cause gastroenteritis infections in humans

**DOI:** 10.1080/19490976.2022.2089007

**Published:** 2022-06-22

**Authors:** Meiying Yan, Yongming Zhou, Yang Cao, Zhenpeng Li, Xin Lu, Bo Pang, Shukun Wang, Biao Kan

**Affiliations:** a State Key Laboratory of Infectious Disease Prevention and Control, National Institute for Communicable Disease Control and Prevention, Beijing, China; b Institute for Acute Communicable Disease Prevention and Control, Yunnan Center for Disease Control and Prevention, Kunming, Yunnan, China

**Keywords:** *Salmonella*, serovar, genome, virulence, gastroenteritis

## Abstract

Some serovars of *Salmonella* are not or rare found to cause salmonellosis in human. In our clinic-based surveillance, three rare *Salmonella* 4,5,12:a:- strains were recovered from three patients with diarrhea. To explore their genetic and epidemiological characteristics and pathogenesis, we conducted whole-genome sequencing, *in vitro* invasion assays in mammalian cells, and *in vivo* virulence assays in an animal model. The three isolates had indistinguishable molecular patterns and similar genome sequences, and clustered together with an isolate from edible fish traded among countries. The isolates had biochemical reactions identical with those of *Salmonella* subspecies *enterica* but belonged to subspecies *salamae* according to genome phylogeny, revealing a new serovar, *S. enterica* subsp. II serovar 4,5,12:a:-. The strains contained multiple virulence genes, elicited temporary bacteremia and enteritidis and caused cell damage in the mouse liver and cecum. This study provides evidence that this new *Salmonella salamae* serovar can infect humans and cause clusters of cases, and whole-genome sequencing detection and surveillance of *Salmonella* can help to accurately define *Salmonella* classification and clonality, improve diagnosis, facilitate outbreak detection and aid in the source tracing of salmonellosis epidemics.

## Introduction

Salmonellosis is one of the common foodborne diseases in human. It is estimated that *Salmonella* causes 78.7 million human infections and 59,000 deaths annually worldwide.^1^ Although more than 2,500 *Salmonella* serovars have been identified, most human infections are caused by a limited number of serovars.^2^ Some uncommon serovars may appear and even cause outbreaks in a region.^3,4^ Outbreaks caused by uncommon serovars may indicate new food contamination and emerging issues with food safety.

The human-restricted serovars *S*. Typhi and *S*. Paratyphi cause an invasive, life-threatening systemic disease, typhoid or enteric fever,^5^ while nontyphoidal serovars (NTSs) usually cause self-limited gastroenteritis, associated with intestinal inflammation and diarrhea.^6^ The virulence genes responsible for invasion, survival, and extraintestinal spread are located in *Salmonella* pathogenicity islands (SPIs), which are considered to represent ‘quantum leaps’ in the evolution of *Salmonella* and play a fundamental role in pathogenesis and host specificity.^7–9^ A variety of fimbrial adhesins are involved in the initiation of contact with host cells, followed by the invasion of nonphagocytic cells, especially epithelial cells of the intestinal mucosa, which is predominantly mediated by effectors encoded by SPI-1 island at early stages of infection. *S. enterica* is a facultative intracellular pathogen that survives in macrophages and is able to proliferate in infected host cells within *Salmonella*-containing vacuoles, where SPI-2 promotes intracellular replication of bacteria, eventually leading to systemic infection.

In our surveillance of pathogens associated with diarrhea, three strains possessing the same antigenic formula as *S*. Fulica were obtained.^10^ No human case of *S*. Fulica infection has been reported previously. To investigate the genetic characteristics, epidemiological significance, and pathogenesis of these strains, we performed comparative genomic and pathogenesis assays in this study.

## Methods

### Ethics statement

All experiments involving animals were performed in accordance with protocols approved by the Animal Care and Use Committee of the National Institute for Communicable Disease Control and Prevention and according to the medical research regulations of the National Health Commission, China.

### Epidemiological data and identification of *Salmonella*

Three *Salmonella* strains identified as *S. enterica* subsp. II serovar 4,5,12:a:- were isolated from three inpatients with diarrhea.^10^ Two of the strains were obtained from two children aged less than 12 months, and the third was from a 65-year-old man; all patients had different disease onset dates (Supplementary Table 1) and had no preexisting diseases. First, the designation as *Salmonella* was confirmed by API 20E biochemical tests (bioMerieux, France) and then the strains were serotyped by slide agglutination according to the Kauffmann-White scheme using *Salmonella* antisera (SSI, Denmark). The antigenic formula 4,5,12:a:- was obtained for which serovars Fulica and Hessarek shared a similar profile but exhibited different biochemical characteristics. To differentiate serovar Fulica from Hessarek, and subspecies *enterica* from *salamae*, certain biochemical tests (Supplementary Table 2) were performed. Antimicrobial susceptibility testing was performed and interpreted according to the CLSI standards.^11^

### Molecular subtyping of *Salmonella*

Pulse-field gel electrophoresis (PFGE) was performed according to the PulseNet International protocol^12^ for *Salmonella*, and the results were analyzed by UPGMA using BioNumerics software (version 2.5, Applied Math, Belgium). Multilocus sequence typing (MLST) of the strains was performed as described on the MLST website (http://mlst.ucc.ie/mlst/dbs/Senterica).

### Whole-genome sequencing and phylogenetic inference

Genomic DNA was prepared from overnight cultures using a Wizard Genomic DNA Purification Kit (Promega, USA) according to the manufacturer’s instructions. Whole-genome sequencing (WGS) was performed using the Illumina HiSeq 2000 platform (BGI, China), and strain 1009S1 was further subjected to PacBio RSII sequencing to obtain the complete genome.^13^ A total of 142 *Salmonella* genomes covering all species/subspecies and 72 serotypes (Supplementary Table 3) were used for genome phylogeny analysis. A core genome of only 791,894 bps with 1,223 genes and 128,888 single-nucleotide polymorphisms (SNPs) was obtained. A maximum likelihood tree based on core-genome SNPs was inferred with IQ-TREE (version 2.0).^14^ The 142 strains included in our study were genetically diverse and belonged to different species, subspecies, and serotypes of *Salmonella*, so no recombinant SNPs were removed for phylogenetic analysis. However, for genome analysis of four *S*. 4,5,12:a:- serovar strains (including the three new strains from the patients in this study and one strain of the same serovar from a Chinese-imported fish in the USA), the nonrecombinant SNPs of the core-genome were used after removing recombinants via ClonalFrameML.^15^ In addition, a heatmap was constructed based on the pangenome (Supplementary Methods). The accession numbers of the strain genome sequences in GenBank are 1009S1: SMDU00000000, 2107S1: PRJNA528152, and 2073S1: PRJNA528161. Virulence factors were identified based on the core dataset in the Virulence Factor Database (VFDB).

### Invasion and cytotoxicity assay

Human epithelial HeLa and Caco-2 and murine macrophage RAW264.7 cells were cultured in high glucose (4.5 g/L) Dulbecco’s modified Eagle’s medium (DMEM) supplemented with 10% heat-inactivated fetal bovine serum (FBS, 20% FBS for Caco-2 cells), 1 mM pyruvate and 2 mM L-glutamine at 37°C with 5% CO_2_. The invasion assay was carried out using a gentamicin protection assay as previously described.^16^ Monolayers of Caco-2 and HeLa cells in 24-well plates were infected at a multiplicity of infection (MOI) of 1:100 with *Salmonella* strains which were subcultured from an overnight culture and grown for 3 h to the logarithmic phase. Two hours postinfection (p.i.), cells were washed three times with PBS, extracellular bacteria were killed by gentamicin (100 µg/ml), and the cells were harvested by the addition of lysis buffer (0.1% SDS and 1% Triton X-100 in PBS). Serial dilutions were plated on LB-agar plates for bacterial colony-forming unit (CFU) enumeration. *Salmonella* invasion was determined based on the number of intracellular *Salmonella* bacteria at 2 h p.i. divided by the inoculated number. For the cytotoxicity assay, RAW264.7 cells grown in 96-well plates were infected at an MOI of 1:10 with *Salmonella* using overnight-grown stationary-phase cultures. Twenty hours postinfection, cell viability (survival and adherent cells) was quantified via a crystal violet dye retention assay in 96-well plates.^16,17^ The adherent cells were stained using 0.2% crystal violet in 20% methanol, released by a 50% ethanol/0.1% acetic acid mixture and quantified by absorbance at 577 nm. *S*. Typhimurium SL1344 was used as the positive control in both assays. Student’s *t* test was used to determine the significance of the differences between different strains, and p <0 .05 was considered to be statistically significant. Each experiment was repeated three times.

### Pathogenesis assays in mouse model

The mouse pathogenesis assay was performed using male BALB/c mice aged 6 to 8 weeks and weighing 18 to 20 g.^18^ The strains were orally administered at a dose of 1.8 × 10^7^ CFUs, and the inoculated mice were examined daily for morbidity and mortality for up to seven days after inoculation. For the analysis of bacterial load in the organs, a total of 10 mice in each group were sacrificed at postinoculation days 1, 2, 3, 5, and 7. The targeted organs were mechanically homogenized to quantify *Salmonella* colonies as CFUs (g tissue^−1^). In addition, the dissected tissues from three mice in each group were subjected to histopathological examination via hematoxylin–eosin staining. All histological slides were evaluated by a veterinary pathologist blinded to the treatment groups to assess the level of tissue damage in the different mouse groups. Images were captured under 20× and 200× magnification using an Olympus BX43 microscope with an Olympus DP21 digital camera system. Histological changes or pathological damage, such as congestion, degeneration, necrosis, proliferation, and inflammation were noted. Representative or typical lesions were imaged from each group and are indicated with arrows in the figure. The degree of tissue damage is expressed in five levels based on a pathological scoring system. No damage was assigned a score of 0; mild damage or a few tissue lesions (<25%) was assigned a score of 1; moderate damage or a moderate number of lesions (25%–50%) was assigned a score of 2; severe damage or a large number of lesions (50%–75%) was assigned a score of 3; and very severe damage or an abundant number of lesions (75%–100%) was assigned a score of 4. To demonstrate the colonization of *S*. 4,5,12:a:- in mouse intestines, we observed the bacterial distribution in a mouse model using bioluminescence imaging technology (Supplementary Methods). An unpaired two-tailed *t* test was used to determine the significance of the differences between colonization data, and *p*<0 .05 was considered to be statistically significant.

### Competition assay in mice

Mutant strains and the wild-type strain were grown separately in LB at 37°C for approximately 18 h. The bacteria were washed in PBS, and each mutant was mixed with the wild-type strain at a concentration of 1 × 10^7^ CFU/ml (2 × 10^7^ CFU/ml total bacterial concentration). Dilutions of this suspension were plated onto LB medium with streptomycin only (to measure total CFU) and LB medium with streptomycin and tetracycline (to quantify ΔLEE mutant CFU) or chloramphenicol (to quantify ΔACE mutant CFU) for enumeration. The exact input ratio of the mutant to wild-type was calculated. A 50 µl sample of the mixed suspension was used to infect female, 6- to 8-week-old streptomycin-pretreated C57BL/6 mice by gavage,^18^ with five mice in each group. After 48 h, the colon, cecum and ileum were recovered and homogenates were plated onto selective media to determine the output ratio of the mutant to wild-type. The competitive index (CI) is defined as the output ratio (mutant/wild type) divided by the input ratio (mutant/wild type). For *in vitro* competition assays, approximately 1 × 10^4^ CFU/ml mutant and 1 × 10^4^ CFU/ml wild type were inoculated into 5 ml of LB and grown at 37°C with shaking for 18 h. The input and output ratios of the mutant to wild-type strains were determined by selective plating as described above. CI values were considered significant if they were below 0.5.

### Antibacterial activity assay

Wild-type and mutant strains were incubated with *Escherichia coli* MG1655 (K12) to assess type 6 secretion system (T6SS)-dependent killing. Overnight cultures of bacteria were washed with PBS, followed by resuspension at 10^10^ CFU/ml. The indicated *S. 4,5,12:a:-* bait strain 1009S1 was mixed with *E. coli* prey at a 1:1 ratio (10 µl of each resuspension), spotted on an LB plate supplemented with 0.05% bile salts (Sigma–Aldrich) and grown at 37°C for 48 h. Spots were serially diluted and plated on MacConkey agar to count the CFU. Bait recovered was calculated as the ratio of total bait CFU divided by total *Salmonella* CFU which was scraped and counted on selective plates. Student’s *t* test was used to determine the significance of the differences between different strains, p <0 .05 was considered to be statistically significant. Each experiment was repeated three times.

## Results

### *Salmonella* serovar 4,5,12:a:- may cause gastroenteritis in humans

All three strains with the antigenic formula 4,5,12:a:- were initially confirmed to be *S. enterica* serovar Fulica, and they were sensitive to 16 tested antimicrobials, including penicillin, cotrimoxazole, fluoroquinolones, cephalosporins, chloramphenicol, and azithromycin. Although the associated patients had different onset dates and lived in different counties/districts, the three strains had the same new sequence type (ST) and indistinguishable PFGE patterns, even when digested with two endonucleases (Supplementary Figure 1), suggesting that these strains had similar genetics. The PFGE data were consistent with the WGS data (presented below), which indicates a common exposure or transmission chain among the patients. No other common pathogenic bacteria or viruses causing diarrhea were isolated from these patients.

### The new serovar 4,5,12:a:- strains have clonal genomes and belong to subspecies II

A complete genome of 4,801,759 bp and 4,413 ORFs was obtained for 1009S1, and these parameters were similar for the other two strains. In these three genomes, the sequences of the genes encoding the O and H antigens were identical for O4,5,12 (*rfb* cluster) and Ha (*filC*), and their positions on the chromosome were similar to those of most other *Salmonella* (Supplementary Figure 2). No *fljB* gene was observed, which further confirmed the antigenic formula 4,5,12:a:-. Twenty-nine SNPs were identified in the core genomes of these strains, 26 of which were located in ORFs, with 11 synonymous and 15 nonsynonymous mutations. Although these strains were collected over a five-month period, the genome comparison together with the identical PFGE patterns strongly supported the clustering of these strains, suggesting the spread of this clone in Yuxi and even a possible outbreak.

**Figure 1.**
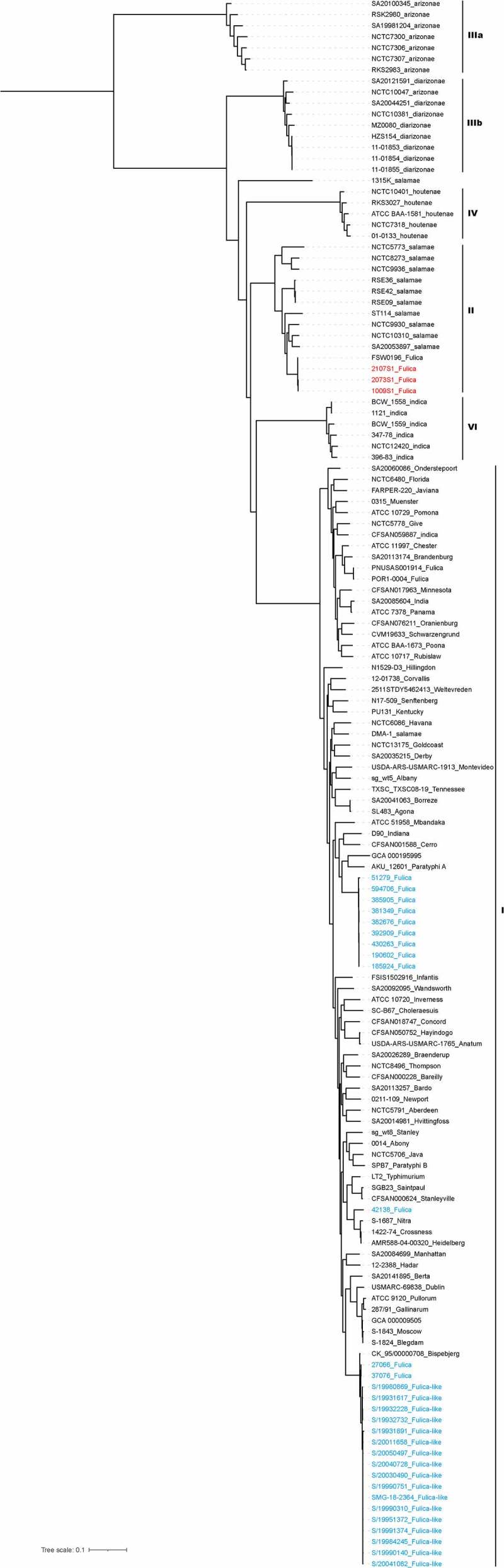
Phylogenetic tree of the genomes of 142 representative *Salmonella* strains of different subspecies and serotypes, including the three *S*. 4,5,12:a:- strains. A maximum likelihood tree was constructed according to the SNPs identified among the genomes of 142 *Salmonella* strains, including the three *S*. 4,5,12:a:- strains sequenced in this study. The three *S*. 4,5,12:a:- strains (red) clustered together to form a separate branch. With the exception of some strains, all isolates were clustered into six groups (I, II, IIIa, IIIb, IV, and VI), which is concordant with their traditional taxonomy. The genome codes are presented as strain code_serovar or subspecies. The strains with the same 4,5,12:a:- antigenic formula, including Fulica and Fulica-like strains, are marked with blue. The dendrogram was constructed with iTOL (https://itol.embl.de).

A maximum likelihood tree showed that the three Yuxi *S*. 4,5,12:a:- strains and FSW0196 clustered together to form a separate branch that shared a recent common ancestor with three subspecies *salamae* strains (Figure 1). Sixty-nine SNPs were identified from the core-genome comparison of the three Yuxi strains and FSW0196; only 12 SNPs and 15 SNPs were observed between FSW0196 and 2107S1 and between FSW0196 and 1009S1. All 10 subspecies *salamae* strains except one grouped together with these three new *S*. 4,5,12:a:- strains to form a separate clade II (Figure 1). The other 124 strains belonging to the other five subspecies formed five clades, with each clade corresponding to its individual subspecies. Three *S. bongori* strains clustered together to form an additional clade V (Supplementary Figure 3). The subspecies *salamae* group was clearly separated from the clade of the subspecies *enterica*, indicating that the newly isolated 4,5,12:a:- strains were genetically different from the known *S*. Fulica and Fulica-like strains (same antigenic formula), of which all 33 strains fell into the subspecies *enterica* clade in the phylogenetic tree. A heatmap based on the pangenome showed a similar genetic result (Supplementary Figure 4). Each subspecies displayed a similar genome composition within the subspecies, and the Yuxi 4,5,12:a:- strains possessed very similar genome compositions and lower genomic diversity with those of the subspecies *salamae*, not with the subspecies *enterica* (Supplementary Figure 3).

Biochemical tests showed that all three *S*. 4,5,12:a:- strains had a profile similar to that of subspecies *enterica* (Supplementary Table 3) and exhibited dual characteristics in the genomic phylogeny analysis and biochemical tests for subspecies identification. Although the 4,5,12:a:- strains have a biochemical phenotype similar to that of subspecies *enterica*, they should be classified as subspecies *salamae* based on their genomes; thus, they are considered a new serotype of *Salmonella, S. enterica* subsp. II serovar 4,5,12:a:-, following the nomenclature of *Salmonella*.

Malonate metabolism is an important biochemical test used to distinguish between subspecies *salamae* and *enterica*. We found high gene diversity and mutations (Supplementary Table 4) in the responsible *mdcABCDEFGH* genes^19^ in *S*. 4,5,12:a:-. In a complementation experiment (Supplementary Methods), only the *mdcCDE*-complemented strain could utilize malonate, whereas strains with individual genes could not (Supplementary Figure 5), indicating that the *mdcCDE* genes play a key role in malonate metabolism and that their mutations cause changes in protein structure or activity, thereby influencing the interaction among these three proteins; eventually, the strain evolves and is unable to metabolize malonate.

### *S.* 4,5,12:a:- strains exhibited virulence in cells

**Figure 2.**
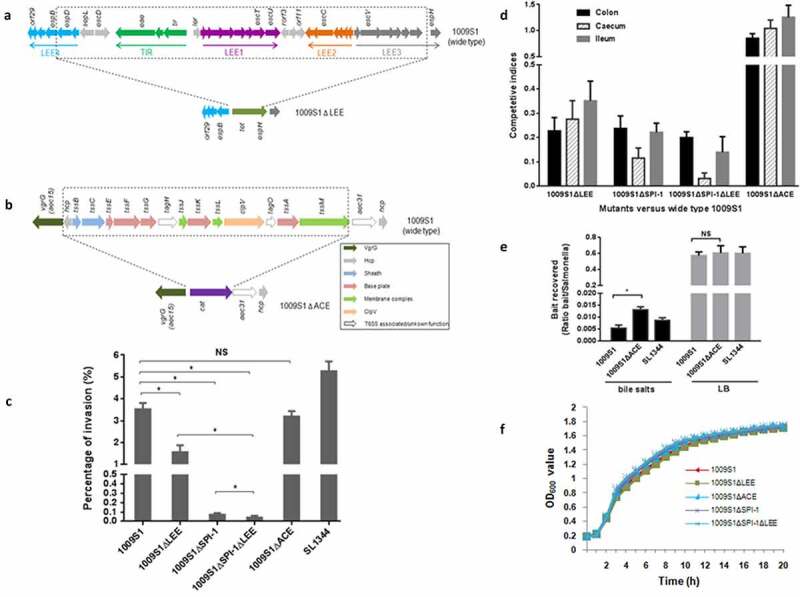
Invasion *in vitro* and colonization *in vivo* assays of *S*. 4,5,12:a:- and the mutants. A is the construction of ΔLEE isogenic strain, most of the LEE genes (dash line box) were deleted and replaced by a *tet* gene, producing the mutant strain 1009S1ΔLEE. B is the construction of ΔACE isogenic strain, most of the ACE genes (dash line box) were deleted and replaced by a *cat* gene, producing the mutant strain 1009S1ΔACE. C: Invasion of HeLa cells by *Salmonella* new serovar and the isogenic strains. It showed the percentage of input values that are gentamicin-protected at 2 h postinfection. Statistical significance was shown based on a Student’s *t* test. NS, not significant, *, P < .05. 1009S1 was the new serovar wide-type strain. 1009S1ΔLEE was the LEE knockout strain of 1009S1. 1009S1ΔSPI-1 was the SPI-1 knockout strain and 1009S1ΔSPI-1ΔLEE was the SPI-1 and LEE double knockout strain. 1009S1ΔACE was the ACE T6SS knockout strain. *S*. Typhimurium SL1344 was the virulence strain, as a positive control. D: Competitive index scores for a number of different mutants. It showed the average CI values of colon, cecum and ileum in different mutants and wide-type treated mouse groups. The CI is defined as the output ratio (mutant/wild-type) divided by the input ratio (mutant/wild-type). CI values are considered significant if they are below 0.5. The black box represents colon, slash box represents cecum and gray box ileum. E: ACE (T6SS) provides competitive advantage against *E. coli in vitro*. The indicated *S*. 4,5,12:a:- 1009S1 bait strain 1009S1was mixed with *E. coli* prey at a 1:1 ratio and incubated for 48 h on an LB agar plate supplemented or not with 0.05% porcine bile salts. Recovered mixtures were plated onto selective media. *S*. Typhimurium SL1344 was used as a positive control. Statistical significance was shown based on a Student’s *t* test corresponding to the values of the wide-type strain (NS, not significant; *P < .05). F: The growth curves of the wide-type 1009S1 and the four isogenic strains in LB. There was no significant growth difference between the five individual strains.

An invasion assay showed that at two hours p.i., approximately 3.5% of wild-type *S*. 4,5,12:a:- strain 1009S1 invaded HeLa cells, reaching 66.92% of the invasive level of SL1344 (Figure 2(c)), which was similar to the invasive level of enteropathogenic *E. coli* (EPEC) for epithelial cells,^20^ while almost no invasion activity was observed with the SPI-1 mutant strain 1009S1ΔSPI-1 (detailed description of the gene deletion protocol in Supplementary Methods). Similar invasive ability changes and levels were observed in the Caco-2 cell line (data not shown). Wild-type 1009S1 displayed significant cytotoxicity to RAW264.7 macrophages compared with that of the isogenic SPI-1 knockout strain (p <0 .05), with slightly less cytotoxicity to RAW264.7 cells similar to that of the virulent strain *S*. Typhimurium SL1344 (p >0 .05, Supplementary Table 5). These results further demonstrated the role of SPI-1 in cell invasion, and indicates the virulence of *S*. 4,5,12:a:-.

### *S.* 4,5,12:a:- strains exhibited pathogenicity in a mouse model

**Figure 3.**
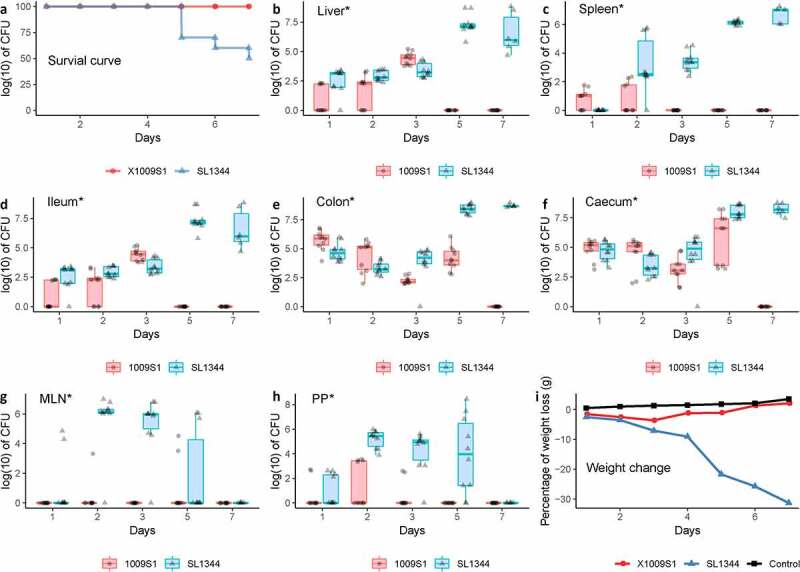
Kinetic analysis of *S*. 4,5,12:a:- and *S*. Typhimurium infection and colonization in the different organs of mice. The data represent mouse survival (a) and CFU/g tissue in the liver (b), spleen (c), ileum (d), cecum (e), colon (f), MLNs (g) and PPs (h) of mice infected with *S*. 4,5,12:a:- strain 1009S1 or *S*. Typhimurium strain SL1344 over seven days. Blue circles represent *S*. 4,5,12:a:- 1009S1-infected mice. Pink triangles represent *S*. Typhimurium SL1344-infected mice. The horizontal line is the mean of each group. * Indicates that 1) the survival rate of 1009S1-infected mice, 2) the number of CFUs recovered from 1009S1-infected mice, and 3) the percentage body weight change in S. 4,5,12:a:--infected mice were significantly different from those of *S*. Typhimurium-infected mice at P <0 .05. The tests were repeated three times.

In the mouse infection experiments, half of the mice infected with SL1344 died on the seventh day after inoculation, whereas all mice infected with 1009S1 survived the seven-day course (Figure 3(a)). A clear and progressive decrease in body weight percentage was observed in the SL1344-infected mice (Figure 3(i)). The 1009S1-infected mice showed a slight decrease in weight that was recovered after four days. Strain 1009S1 colonized the spleen and liver for one and two days, respectively, indicating a poorer colonization ability than that of SL1344 (Figure 3(b)). However, the results still indicated that the *S*. 4,5,12:a:- strain invaded the blood, causing systemic infection in the mouse model.

High burdens of 1009S1 and SL1344 were detected in the ileum, cecum, and colon (Figure 3(d-f)). 1009S1 colonized these organs for a shorter period than SL1344. Bacterial levels in the ileum were significantly lower than those in both the cecum and colon throughout the course of infection (1,000–10,000-fold, P <0 .05, Figure 3(d-f)). No significant difference was observed between cecum and colon colonization in 1009S1-infected mice. We also observed the intestinal colonization of bacteria in the mice after gavage with lux-labeled strains (Supplementary Figure 6), similar to the results of the bacterial culture experiments.

In the mesenteric lymph nodes (MLNs) of mice, strain 1009S1 recovered within three days after inoculation, and SL1344 recovered within at least five days after inoculation (Figure 3(g)). In the Peyer’s patches (PPs), both strains were isolated within five days after inoculation, but 1009S1 was intermittently recovered (Figure 3(h)). The colonization of SL1344 increased significantly starting on postinfection day 2 (1,500-fold, P <0 .05), while 1009S1 exhibited a weaker colonization ability. Similar colonization levels were observed among the organs for 1009S1.

### *S.* 4,5,12:a:- caused mild pathological manifestations in the digestive organs of mice

**Figure 4.**
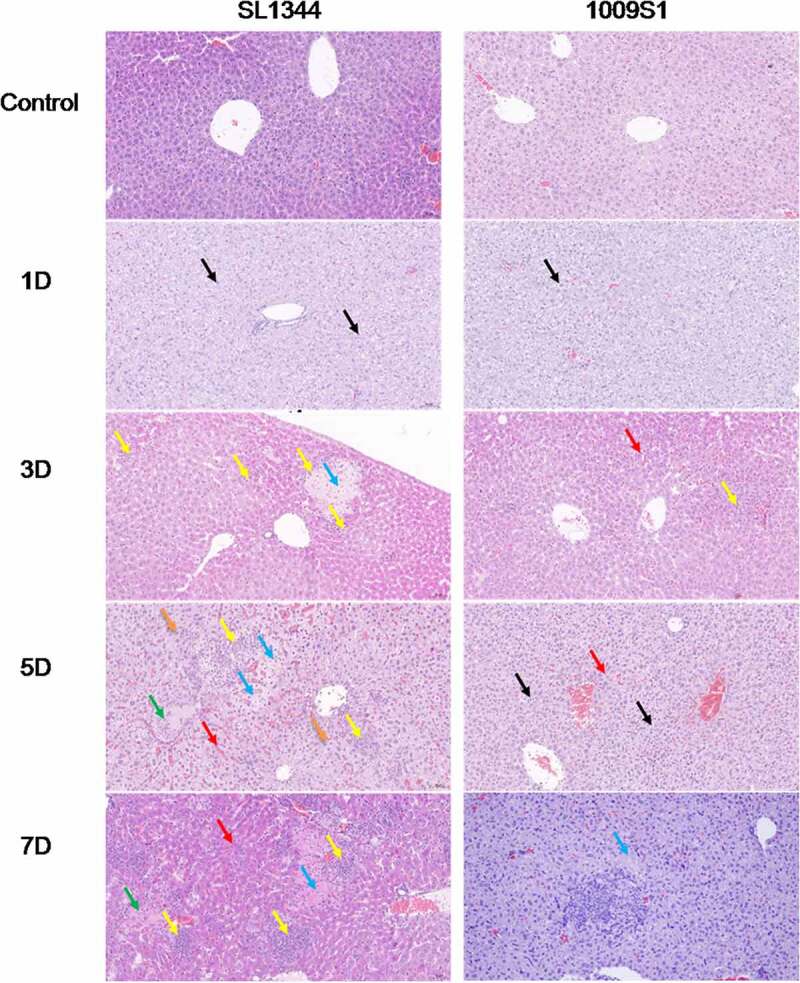
Histopathological examination of the liver in the mouse model. The dissected liver samples were collected from mice infected with strain 1009S1 or SL1344 at different time points after inoculation. The figure shows representative mice with typical histopathological damages for each tested strain at postinfection days 1, 3, 5 and 7. Uninfected mice were used as the normal (negative) controls. For strain 1009S1, the image on day 7 shows that one of the 10 mice infected by 1009S1 displayed focal hepatocyte necrosis, while the other mice appeared normal. The black arrow indicates watery degeneration of hepatocytes, the yellow arrow indicates neutrophil infiltration, the red arrow indicates congestion of hepatic sinus, the blue arrow indicates hepatocyte necrosis, the green arrow indicates inflammatory necrotic material blocking the central vein, and the brown arrow indicates hepatic microvesicular steatosis. 100× magnification.

**Figure 5.**
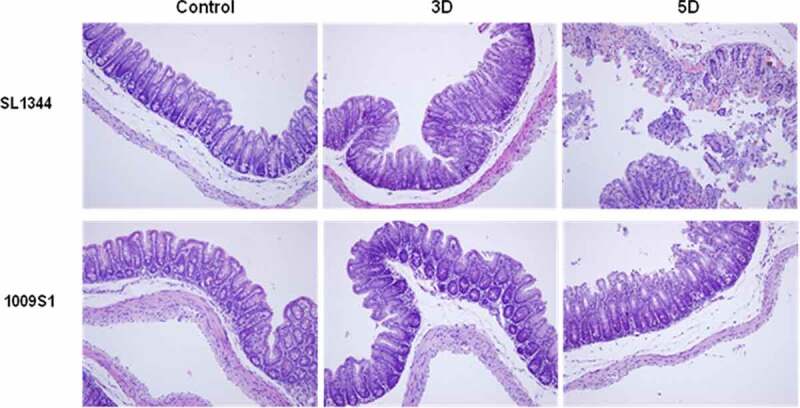
Histopathological examination of the cecum in the mouse model. The dissected ceca were collected from mice infected with *S*. 4,5,12:a:- strain 1009S1 or *S*. Typhimurium strain SL1344 at different time points post infection and stained with hematoxylin-eosin for histopathological examination under a microscope. The figure shows one representative sample from a mouse with typical histopathological damage for each tested strain at 3 and 5 days post-infection. Other histopathological damage of the cecum is not shown here due to the similarity of the pathological changes. Uninfected mice were used as a normal negative control. The magnification is 200 × .

The liver displayed pathological changes on the first day of infection with the control strain SL1344. A composite pathology score (see details in the methods) is shown in Supplementary Figure 7. The pathological changes increased in severity, from hepatocyte centrilobular hydropic degeneration to diffuse necrosis, as the infection progressed (Figure 4 and Supplementary Figure 7A). Changes in mice infected with 1009S1 appeared at only the early stage of infection, presenting as mild hepatocyte hydropic degeneration on postinfection days 2 and 3. Subsequently, the changes disappeared and the tissue recovered to a normal preinfection state, with the exception of that in one mouse that developed focal hepatocyte necrosis on the seventh day. The changes and levels of pathology in the cecum followed a similar course to those in the liver. The intestinal epithelial cells of SL1344-infected mice were extensively exfoliated and necrotic (Figure 5), with gradually increasing severity as the infection progressed (Figure 5 and Supplementary Figure 7B). Most 1009S1-infected mice appeared normal on postinfection day 7, except one mouse presenting with mild inflammation in the late stage of infection.

### Virulence genes in *S.* 4,5,12:a:- strains

SPIs 1–3, 5, 6, 9, 11, 12, 16 and 19 and the partial genes of SPI-18 and *hlyE* were present in all *S*. 4,5,12:a:- strains. All SPI-1 and SPI-2 genes and 10 effectors, including *sipA, sipB, sipC, sopA, sopD, sopE2*, and *sptP*, translocated by the SPI-1 type 3 secretion system (T3SS) were also detected in these strains. Similarly, most of the SPI-2 translocated effectors, including *spiC, sseF, sseG, sifA, sifB, sspH2, sseJ, sseL, sseK2* and *pipB2*, were present in the strains. However, other effectors including *avrA, sopB, sopE, slrP, sspH1, sopD2, sopF, sseK1, sseK3, gogB, pipB*, and *sseI* were absent in these strains. Universal fimbrial clusters (*bcf, csg, fim, stb*, and *sth*), one ‘typhoid’ fimbrial operon *sta*, and an invasion gene *pagN* were present in the genomes of these strains. Some nonfimbrial adherence determinants, including *misL, ratB*, and *sinH*, were also present. Other virulence genes, such as *phoPQ, mig14*, and *mgtBC*, were also found. Additionally, the locus of enterocyte effacement (LEE) of *E. coli* O157:H7,^21^ and ACE T6SS of *E. coli* were found in these strains, as well as *eae* and *usp*.

### The LEE of *S.* 4,5,12:a:- was involved in cell invasion and colonization in a mouse model, and ACE had antibacterial activity *in* *vitro*

The LEE is required for attachment/effacement (A/E) lesion formation and is the most intensively studied of all virulence factors in A/E-inducing *E. coli*. The structure and sequence of the locus is nearly the same as that of *S*. Sofia (*S. enterica* subspecies *salamae* serovar 1,4,12,27:b:-),^21^ in which the operons TIR and LEE4 are reverse complemented and moved to the 5’ end of LEE1, rather than the more typical arrangement at the 3’ end of LEE3 in *Escherichia* and *Citrobacter rodentium*.^22^ The percentage identity between the two loci was 99.6%, and most of the genes in the LEE had no SNPs or mutations. To determine the function of the *E. coli* O157:H7 LEE-related locus in the *S*. 4,5,12:a:- strain, we deleted most LEE genes (1009S1ΔLEE, Figure 2(a)), and then performed an *in vitro* invasion assay using HeLa cells and a CI experiment in a streptomycin-pretreated mouse model. The results showed that the 1009S1ΔLEE displayed slightly reduced (2.3 fold) invasion ability in HeLa cells (Figure 2(c)) and significantly decreased ΔLEE CFU recovered from the mouse colon, cecum, and ileum (Figure 2(d), CI < 0.5) compared with those of the wild-type *S*. 4,5,12:a:- strain, and a similar result was observed in Caco-2 cells, indicating LEE involving in the pathogenesis of *S*. 4,5,12:a:-.

To determine whether the two loci LEE and SPI-1 together contribute to the invasion and colonization abilities of the new serovar, we constructed a ΔSPI-1ΔLEE double mutant (Supplementary Methods). The invasion level of the ΔSPI-1ΔLEE strain in HeLa cells was dramatically reduced to 0.04%, and the invasion level of ΔSPI-1 was significantly reduced to 0.07% compared with that of the wild-type *S*. 4,5,12:a:- strain (Figure 2(c)). In mouse competition assays, significantly decreased ΔSPI-1ΔLEE CFU were recovered from the mouse intestine compared with those from the intestine of mice infected by either the wild-type *S*. 4,5,12:a:- strain or the single-gene deletion strain, ΔLEE and ΔSPI-1. The CIs for the ΔSPI-1ΔLEE, ΔSPI-1, and ΔLEE mutants were 0.03–0.2, 0.12–0.24, and 0.23–0.35, respectively, approximately 3–30-fold decrease in colonizing level comparing to the wild-type strain, especially for ΔSPI-1ΔLEE cecum colonization (Figure 2(d)). These results demonstrated the contribution of the LEE to the colonization of *S*. 4,5,12:a:- in a mouse model and indicated that it may collaborate with SPI-1 to facilitate bacterial invasion.

In addition, to identify the function of the ACE T6SS of *E. coli* in the *S*. 4,5,12:a:- strain, most of the ACE (T6SS) island genes were deleted in frame, producing the mutant strain 1009S1ΔACE (Figure 2(b)), which was subjected to invasion assays *in vitro* and CI experiments in mice. The results showed no significantly decreased invasion of the ΔACE mutant to HeLa and Caco-2 cells compared with that of the wild-type *S*. 4,5,12:a:- strain, and there were no differences in the colonization abilities of the wild-type and ΔACE isogenic strains (Figure 2(c) (p >0 .05) and Figure 2(e), CI > 0.5). There was no growth difference between the wild-type *S*. 4,5,12:a:- strain and the ΔLEE, and ΔACE strains in LB broth (Figure 2(f)), and the CI values of the wild-type and mutant strains determined *in vitro* were all above 0.5 (between 0.976 and 1.045).

In many gram-negative bacteria, the T6SS is dedicated to target and kill other bacteria to compete for niches and resources.^23–26^ In addition to the core set of 13 genes required for T6SS assembly and function, the *S*. 4,5,12:a:- ACE locus contains two *hcp* genes (Figure 2(b)). To determine whether the ACE (T6SS) of *S*. 4,5,12:a:- can kill other bacteria, we conducted an antibacterial activity assay *in vitro* using *E. coli* MG1655 as prey. When we mixed the wide-type or ΔACE bacterial strains with MG1655 on plates containing bile salts, *S*. 4,5,12:a:- outcompeted MG1655 *in vitro* in an ACE T6SS-dependent manner (p <0 .05, Figure 2(e)), while no significant change in killing was observed between the wide-type and ΔACE strains on an LB plate without bile salts, which further confirmed that bile salts increase the SPI-6 T6SS antibacterial activity of *Salmonella*.^27^

## Discussion

We reported that a new serovar, *S*. 4,5,12:a:-, caused a local gastroenteritis case cluster. To our knowledge, this is the first report of the variant of serovar 4,5,12:a:-, which genetically belongs to subspecies II, causing infection and a probable outbreak in humans. The cell invasion assay and animal infection model were used to further elucidate the pathogenesis and the function of the LEE in bacterial colonization.

For some *Salmonella* strains, genome-based phylogeny and serotyping are incompatible for classification. Serotyping identifies only lipopolysaccharide and flagellar epitope variance, whereas burst grouping based on housekeeping gene sequences^28,29^ have revealed the natural population structure of *Salmonella*. Gene recombination is frequent within bacteria. In *Salmonella*, genes encoding antigenic epitopes also exhibit horizontal exchange and homologous recombination among genetic lineages.^28,30,31^ Current molecular serotyping tools, such as SeqSero, also predict isolates with the antigenic formula 4,5,12:a:-. Although genome-based predictions of *Salmonella* serotypes are increasingly being used worldwide^28,32,33^ and are more reliable than phenotyping, they all rely on similar antigenic markers or partial regions of genomes. WGS revealed that the three 4,5,12:a:- isolates were closely related to subspecies *salamae* strains and distantly related to subspecies *enterica* and other subspecies. Combining phylogenetic characteristics and serology-based serotyping results, the three 4,5,12:a:- isolates were reassigned as *Salmonella salamae* 4,5,12:a:-. These cases provide evidence of the nonconformity between serotyping and genomic phylogeny in *Salmonella*, which is mainly due to differences in partial biological molecular characteristics and genomic SNP/recombination. The contradictory subspecies typing results between genomic and phenotypic classifications of the 4,5,12:a:- strains highlight the need for improvement in current *Salmonella* serotyping approaches.

Fimbriae and pili play central roles in bacterial adherence and colonization.^34^ The genome sequences of the *S*. 4,5,12:a:- isolates revealed the presence of several universal SPIs, including intact SPI-1 and SPI-2, and the majority of effectors translocated in the two systems, indicating that *S*. 4,5,12:a:- has two functionally distinct T3SSs that enable the invasion of epithelial cells by bacteria and facilitate the replication of bacteria within macrophages. This hypothesis was confirmed by the cell invasion tests in Caco-2, HeLa and macrophage cells, and competition assays and mouse infection models in this study. In addition to virulence genes, a *fimH* allele, famH80, which has recently been identified as human-prone and human-specific,^35^ was also found in this serovar. Furthermore, we found that the *S*. 4,5,12:a:- strains harbored an LEE locus, which was previously observed to be specific to *S. salamae*.^21^ All of these characteristics illustrate that *S. salamae* shares some genetic characteristics with *E. coli*, especially those related to extracellular pathogenicity. Additionally, it was demonstrated that 4,5,12:a:- belongs to subspecies II, not I. All of the above factors may play roles in the pathogenicity and/or in the invasive characteristics of *S*. 4,5,12:a:-, possibly by contributing to tissue tropism and/or colonization.

The human pathogen EHEC, EPEC, and the mouse pathogen *C. rodentium* (CR) possess the LEE locus, which encodes a type III secretion system that injects effectors into host cells and is sufficient for inducing A/E lesions, actin-rich pedestals.^36,37^ The LEE contains 41 genes and all of LEE genes are required for full virulence of *C. rodentium* in mice.^22^ The *S*. 4,5,12:a:- contained an intact LEE locus. When individually deleting LEE and SPI-1 in the new serovar, the ability of bacteria to invade cells and colonize the intestinal tract of mice decreased, but the reduction level of the ΔLEE strain was lower than that of the ΔSPI-1 strain. When LEE and SPI-1 were simultaneously deleted, the ability of bacteria to invade cells and colonize the mouse intestine is much lower than that of ΔLEE strain, slightly lower than ΔSPI-1 strain (Figure 2), indicating that LEE, like SPI-1, mediates bacterial invasion to epithelial cells, and which was enhanced with the presence of SPI-1, that is, SPI-1 and LEE together contribute to the colonization and invasion of the new serovar strain in the enteric cells, suggesting a synergistic effect on bacterial virulence in cells where SPI-1 played a major role.

The genetic structure of ACE T6SS in *S*. 4,5,12:a:- was different from SPI-6 T6SS of *S*. Typhimurium,^26^ more similar to SPI-19 of *S. enterica* subsp. *salamae* ST114 (an *in silico* analysis),^38^ thereby a new SPI-19 type found in *Salmonella*. The antibacterial activity assay revealed that *S*. 4,5,12:a:- killed intestinal bacteria in an ACE T6SS-dependent manner in the presence of bile salts in an *in vitro* settings, providing evidence for the bactericidal activity of the new SPI-19 island that may contribute to the bacterial invasion of gut during infection. However, the new SPI-19 has no significant effect on the bacterial colonization in the early stage of infection (Figure 2(d)) and this may be due to the mouse used in this study was the streptomycin-pretreated SPF mouse.^27^ Further study will be needed to clarify it.

The presence of SPI-2 facilitates the survival and subsequent spread of bacteria to organs in the mouse model,^39–41^ as shown *in viv*o (Figure 3), however, genetic diversity or other unknown factors may attenuate the strain, resulting in transient bacteriemia and mild symptoms. Compared with other serotypes of NTS that commonly cause gastroenteritis, *S*. 4,5,12:a:- is most similar to invasive typhoidal *Salmonella* in the settings of bloodstream invasion and causing systemic infection in other organs (though persisting for only 2 days in the animal model), suggesting the new threat of this strain as a foodborne disease and the need for additional surveys on the infection. Because of the lack of timely investigations in these cluster cases and the lack of collection of food and environmental samples, no exact link between the illness and food consumption could be established. One additional case caused by *S*. 4,5,12:a:- was detected in Shanghai city (a long distance from Yuxi) in the following year, but the PFGE pattern of the strain was different from that of *S*. 4,5,12:a:-, indicating another infection event in China.

The phylogenetic tree also showed that the three new serovar strains clustered together with an isolate from imported edible fish from China in the USA, supporting that this strain was also *S*. 4,5,12:a:- and raising concern about their sources and the possible spread of this genetic clone. WGS analysis can promote accurate pathogen identification, serotyping, and outbreak investigation.^42–45^ WGS surveillance of *Salmonella* will facilitate the timely identification of potentially emerging and locally important clusters of cases and will help to promote improved practices on a global basis.

## Supplementary Material

Supplemental Material

## Data Availability

The authors confirm that the data supporting the findings of this study are available within the article and its supplementary materials.
