# Supplementary material for: Salmonella enterica subsp. II serovar 4,5,12:a:- may cause gastroenteritis infections in humans
Source: Gut Microbes. 2022 Jun 22;14(1):2089007. doi: 10.1080/19490976.2022.2089007 (PMC9235882; doi:10.1080/19490976.2022.2089007)
Supplement: Supplemental Material [file KGMI_A_2089007_SM0127.docx]

**Supplementary**

**Supplementary Table 1.** The epidemiological and clinical data of the three patients infected by *S*. 4,5,12:a:-

| **Case** | **Strain ID** | **Epidemiological data** | | | |  | **Symptoms** | | |  | **Clinical detection** | | |  | **Antibiotic**  **treatment** | **Hospitalization** |
| --- | --- | --- | --- | --- | --- | --- | --- | --- | --- | --- | --- | --- | --- | --- | --- | --- |
|  |  | **Age** | **Sex** | **Address** | **Onset** |  | **Diarrhea**  Times/day | **Fever** | **Vomiting** |  | **Blood test** | | **Stool** |  |  |  |
| Case1 | 1009S1 | 5 m | male | Jiangchuan county | Jul. 7 |  | 7-8 | >39°C,  2-day fever | + |  | | PL ↑* | Mucous,pus; WBCs 10-15/hp |  | Amoxicillin & clavulanic acid, I.V. | 10d |
| Case2 | 2073S1 | 10m | male | Hongta district | Oct. 16 |  | 5-6 | >38.5°C,  1-day fever | + |  | PL ↑* | | Mucous; WBCs 5-10/hp |  | Ceftriaxone, I.V. | 5d |
| Case3 | 2107S1 | 65y | male | Hongta district | Dec. 12 |  | 5-6 | ND | - |  | - | | watery;  WBCs 5-10/hp |  | Ceftriaxone | 7d |

* peripheral leukocytes increased. I.V.: intravenous injection. ND: not determined.

**Supplementary Table 2.** Biochemical tests for the differentiation of the new *Salmonella* serovar

| Biochemical tests | *S.*Fulica | *S.*Hessarek | II 4,5,12:a:- | Characters | subsp. *enterica* | subsp. *salamae* | II 4,5,12:a:- |
| --- | --- | --- | --- | --- | --- | --- | --- |
| Rhamnose | - | + | - | ONPG  (2 h) | - | - | - |
| Dulcitol | - | + | - | Malonate | - | + | - |
| Trehalose | - | + | - | Gelatinase | - | + | - |
| Simmons citrate | - | + | - | Sorbitol | + | + | + |
| L(+) tartrate | - | + | - | Growth with KCN | - | - | - |
| Mucate | - | + | - | Galacturonate | - | + | - |
| H_2_S | - | + | - | Salicin | - | - | - |
| Tetrathionate-  reductase | - | + | - | Lactose | - | - | - |

**Supplementary Table 3.** The *Salmonella* genomes analyzed in this study

| **Strain** | **Serotype/Formula** | **Source of isolation** | **Isolation year** | **RefSeq** |
| --- | --- | --- | --- | --- |
| *Salmonella* subsp.enterica |  |  |  |  |
| NCTC5791 | Aberdeen |  | 1885/1939 | [NZ_LS483453.1](https://www.ncbi.nlm.nih.gov/nuccore/NZ_LS483453.1) |
| 0014 | Abony |  |  | [CP007534.1](http://www.ncbi.nlm.nih.gov/nuccore/605546632) |
| SL483 | Agona | Human | 2003 | [NC_011149.1](https://www.ncbi.nlm.nih.gov/nuccore/NC_011149.1) |
| sg_wt5 | Albany | Wet market | 2016 | [NZ_CP036165.1](https://www.ncbi.nlm.nih.gov/nuccore/NZ_CP036165.1) |
| USDA-ARS-USMARC-1765 | Anatum | Human stool | 2006 | [NZ_CP014659.2](https://www.ncbi.nlm.nih.gov/nuccore/NZ_CP014659.2) |
| SA20113257 | Bardo | Meleagris | 2011 | [NZ_CP019404.1](https://www.ncbi.nlm.nih.gov/nuccore/NZ_CP019404.1) |
| CFSAN000228 | Bareilly |  |  | [NZ_CP039498.1](https://www.ncbi.nlm.nih.gov/nuccore/NZ_CP039498.1) |
| SA20141895 | Berta |  |  | [NZ_CP030005.1](https://www.ncbi.nlm.nih.gov/nuccore/NZ_CP030005.1) |
| CK_95/00000708 | Bispebjerg | Turtle | 1995 | SAL_NB8020AA_AS |
| S-1824 | Blegdam |  |  | [NZ_CP019406.1](https://www.ncbi.nlm.nih.gov/nuccore/NZ_CP019406.1) |
| SA20041063 | Borreze |  | 1990 | [NZ_CP019407.1](https://www.ncbi.nlm.nih.gov/nuccore/NZ_CP019407.1) |
| SA20026289 | Braenderup |  |  | [NZ_CP022490.1](https://www.ncbi.nlm.nih.gov/nuccore/NZ_CP022490.1) |
| SA20113174 | Brandenburg |  |  | [NZ_CP029999.1](https://www.ncbi.nlm.nih.gov/nuccore/NZ_CP029999.1) |
| CFSAN001588 | Cerro | Bovine stool | 2004 | [NZ_CP012833.1](https://www.ncbi.nlm.nih.gov/nuccore/NZ_CP012833.1) |
| ATCC 11997 | Chester |  |  | [NZ_CP019178.1](https://www.ncbi.nlm.nih.gov/nuccore/NZ_CP019178.1) |
| SC-B67 | Choleraesuis | Human blood | 2002 | [NC_006905.1](https://www.ncbi.nlm.nih.gov/nuccore/NC_006905.1) |
| CFSAN018747 | Concord |  |  | [NZ_CP028196.1](https://www.ncbi.nlm.nih.gov/nuccore/NZ_CP028196.1) |
| 12-01738 | Corvallis | Wild bird | 2012 | [NZ_CP027677.1](https://www.ncbi.nlm.nih.gov/nuccore/NZ_CP027677.1) |
| 1422-74 | Crossness | Sewage | 1973 | [NZ_CP019408.1](https://www.ncbi.nlm.nih.gov/nuccore/NZ_CP019408.1) |
| SA20035215 | Derby |  |  | [NZ_CP022494.1](https://www.ncbi.nlm.nih.gov/nuccore/NZ_CP022494.1) |
| USMARC-69838 | Dublin | Bovine lymph node | 2012 | [NZ_CP032449.1](https://www.ncbi.nlm.nih.gov/nuccore/NZ_CP032449.1) |
| P125109 | Enteritidis | Human | 1991 | [NC_011294.1](http://www.ncbi.nlm.nih.gov/nuccore/207855516) |
| NCTC6480 | Florida | Human | 1942 | [NZ_LS483454.1](https://www.ncbi.nlm.nih.gov/nuccore/NZ_LS483454.1) |
| 185924 | Fulica | Human | 2015 | SAL_BB4750AA_AS |
| 190602 | Fulica | Human | 2015 | SAL_CB2024AA_AS |
| 27066 | Fulica | Human | 2014 | SAL_IA9120AA_AS |
| 37076 | Fulica | Human | 2014 | SAL_EA7048AA_AS |
| 381349 | Fulica | Human | 2017 | SAL_JB9567AA_AS |
| 382676 | Fulica | Food | 2017 | SAL_JB5399AA_AS |
| 385905 | Fulica | Food | 2017 | SAL_YA5779AA_AS |
| 392909 | Fulica | Food | 2017 | SAL_YA5813AA_AS |
| 42138 | Fulica | Human | 2014 | SAL_EA8028AA_AS |
| 430263 | Fulica | Food | 2017 | SAL_YA5818AA_AS |
| 51279 | Fulica | Human | 2014 | SAL_BA4365AA_AS |
| 594706 | Fulica | Human | 2018 | SAL_HB4468AA_AS |
| PNUSAS001914 | Fulica |  | 1997 | SAL_LA0610AA_AS |
| POR1-0004 | Fulica | Swine feces | 2013 | SAL_FA2237AA_AS |
| S/19931617 | Fulica-like | Harborporpoise | 1993 | SAL_OB3935AA_AS |
| S/19931891 | Fulica-like | Harborporpoise | 1993 | SAL_VA5293AA_AS |
| S/19932228 | Fulica-like | Harborporpoise | 1993 | SAL_TA9882AA_AS |
| S/19932732 | Fulica-like | Harborporpoise | 1993 | SAL_OB4253AA_AS |
| S/19951372 | Fulica-like | Harborporpoise | 1995 | SAL_TA9881AA_AS |
| S/19980869 | Fulica-like | Harborporpoise | 1998 | SAL_VA4503AA_AS |
| S/19984245 | Fulica-like | Harborporpoise | 1998 | SAL_VA4456AA_AS |
| S/19990140 | Fulica-like | Harborporpoise | 1999 | SAL_VA4487AA_AS |
| S/19990310 | Fulica-like | Harborporpoise | 1999 | SAL_VA5300AA_AS |
| S/19990751 | Fulica-like | Harborporpoise | 1999 | SAL_VA4510AA_AS |
| S/19991374 | Fulica-like | Harborporpoise | 1993 | SAL_OB4254AA_AS |
| S/20011658 | Fulica-like | Harborporpoise | 2001 | SAL_OB4255AA_AS |
| S/20030490 | Fulica-like | Harborporpoise | 2003 | SAL_OB4528AA_AS |
| S/20040728 | Fulica-like | Harborporpoise | 2004 | SAL_VA4471AA_AS |
| S/20041082 | Fulica-like | Harborporpoise | 2004 | SAL_DB4266AA_AS |
| S/20050497 | Fulica-like | Harborporpoise | 2005 | SAL_DB4264AA_AS |
| SMG-18-2364 | Fulica-like |  | 2018 | SAL_HB1568AA_AS |
| 287/91 | Gallinarum | Chicken |  | [NC_011274.1](https://www.ncbi.nlm.nih.gov/nuccore/NC_011274.1) |
| NCTC5778 | Give |  | 1885/1939 | [NZ_LS483463.1](https://www.ncbi.nlm.nih.gov/nuccore/NZ_LS483463.1) |
| NCTC13175 | Goldcoast |  | 1800/2017 | [NZ_LR134158.1](https://www.ncbi.nlm.nih.gov/nuccore/NZ_LR134158.1) |
| 12-2388 | Hadar | Human |  | [NZ_CP038595.1](https://www.ncbi.nlm.nih.gov/nuccore/NZ_CP038595.1) |
| NCTC6086 | Havana | Environmental | 1900/1940 | [NZ_LR134187.1](https://www.ncbi.nlm.nih.gov/nuccore/NZ_LR134187.1) |
| CFSAN050752 | Hayindogo | Anaheim pepper | 2016 | [NZ_CP017719.1](https://www.ncbi.nlm.nih.gov/nuccore/NZ_CP017719.1) |
| AMR588-04-00320 | Heidelberg | Chicken feces | 2013 | [NZ_CP016569.1](https://www.ncbi.nlm.nih.gov/nuccore/NZ_CP016569.1) |
| N1529-D3 | Hillingdon |  |  | [NZ_CP019410.1](https://www.ncbi.nlm.nih.gov/nuccore/NZ_CP019410.1) |
| SA20014981 | Hvittingfoss | Crocodylus | 2001 | [NZ_CP022503.1](https://www.ncbi.nlm.nih.gov/nuccore/NZ_CP022503.1) |
| SA20085604 | India |  | 2008 | [CP022015.1](https://www.ncbi.nlm.nih.gov/nuccore/CP022015.1) |
| D90 | Indiana | Chicken | 2013 | [NZ_CP022450.1](https://www.ncbi.nlm.nih.gov/nuccore/NZ_CP022450.1) |
| FSIS1502916 | Infantis |  | 2015 | [NZ_CP016408.1](https://www.ncbi.nlm.nih.gov/nuccore/NZ_CP016408.1) |
| ATCC 10720 | Inverness |  |  | [NZ_CP019181.1](https://www.ncbi.nlm.nih.gov/nuccore/NZ_CP019181.1) |
| NCTC5706 | Java |  |  | [NZ_LT571437.1](https://www.ncbi.nlm.nih.gov/nuccore/NZ_LT571437.1) |
| FARPER-220 | Javiana | Liver and spleen of chicken [broiler] | 20117 | [NZ_CP038233.1](https://www.ncbi.nlm.nih.gov/nuccore/NZ_CP038233.1) |
| PU131 | Kentucky | Humanstool | 2013 | [NZ_CP026327.1](https://www.ncbi.nlm.nih.gov/nuccore/NZ_CP026327.1) |
| SA20084699 | Manhattan |  |  | [NZ_CP022497.1](https://www.ncbi.nlm.nih.gov/nuccore/NZ_CP022497.1) |
| ATCC 51958 | Mbandaka |  |  | [NZ_CP019183.1](https://www.ncbi.nlm.nih.gov/nuccore/NZ_CP019183.1) |
| CFSAN017963 | Minnesota | Mangoes | 2014 | [NZ_CP017720.1](https://www.ncbi.nlm.nih.gov/nuccore/NZ_CP017720.1) |
| USDA-ARS-USMARC-1913 | Montevideo | Bovine peripheral lymph node | 2011 | [NZ_CP025278.1](https://www.ncbi.nlm.nih.gov/nuccore/NZ_CP025278.1) |
| S-1843 | Moscow |  |  | [NZ_CP019415.1](https://www.ncbi.nlm.nih.gov/nuccore/NZ_CP019415.1) |
| 0315 | Muenster | Bovine stool | 2001 | [NZ_CP019198.1](https://www.ncbi.nlm.nih.gov/nuccore/NZ_CP019198.1) |
| 0211-109 | Newport | Bovine stool | 2002 | [NZ_CP012598.1](https://www.ncbi.nlm.nih.gov/nuccore/NZ_CP012598.1) |
| S-1687 | Nitra |  |  | [NZ_CP019416.1](https://www.ncbi.nlm.nih.gov/nuccore/NZ_CP019416.1) |
| SA20060086 | Onderstepoort |  | 1998 | [NZ_CP022034.1](https://www.ncbi.nlm.nih.gov/nuccore/NZ_CP022034.1) |
| CFSAN076211 | Oranienburg | Raw pecans |  | [NZ_CP033344.1](https://www.ncbi.nlm.nih.gov/nuccore/NZ_CP033344.1) |
| ATCC 7378 | Panama | Human |  | [NZ_CP012346.1](https://www.ncbi.nlm.nih.gov/nuccore/NZ_CP012346.1) |
| AKU_12601 | Paratyphi A | Human | 2002 | NC_011147.1 |
| SPB7 | Paratyphi B | Human | 1990s | [NC_010102.1](http://www.ncbi.nlm.nih.gov/nuccore/161612313) |
| ATCC 10729 | Pomona | Environmental |  | [NZ_CP019186.1](https://www.ncbi.nlm.nih.gov/nuccore/NZ_CP019186.1) |
| ATCC BAA-1673 | Poona | Iguana | 1997 | [NZ_CP019189.1](https://www.ncbi.nlm.nih.gov/nuccore/NZ_CP019189.1) |
| ATCC 9120 | Pullorum | Human |  | [NZ_CP012347.1](https://www.ncbi.nlm.nih.gov/nuccore/NZ_CP012347.1) |
| ATCC 10717 | Rubislaw |  |  | [NZ_CP019192.1](https://www.ncbi.nlm.nih.gov/nuccore/NZ_CP019192.1) |
| SGB23 | Saintpaul | Wet market | 2016 | [NZ_CP023166.1](https://www.ncbi.nlm.nih.gov/nuccore/NZ_CP023166.1) |
| CVM19633 | Schwarzengrund | Dehydrated chili | 2002 | [NC_011094.1](https://www.ncbi.nlm.nih.gov/nuccore/NC_011094.1) |
| N17-509 | Senftenberg |  | 2017 | [NZ_CP026379.1](https://www.ncbi.nlm.nih.gov/nuccore/NZ_CP026379.1) |
| sg_wt8 | Stanley | Wet market | 2016 | [NZ_CP036167.1](https://www.ncbi.nlm.nih.gov/nuccore/NZ_CP036167.1) |
| CFSAN000624 | Stanleyville |  | 1988 | [NZ_CP017723.1](https://www.ncbi.nlm.nih.gov/nuccore/NZ_CP017723.1) |
| TXSC_TXSC08-19 | Tennessee | Fishmeal | 2004 | [NZ_CP007505.1](https://www.ncbi.nlm.nih.gov/nuccore/NZ_CP007505.1) |
| NCTC8496 | Thompson |  | 1800/1951 | [NZ_LS483493.1](https://www.ncbi.nlm.nih.gov/nuccore/NZ_LS483493.1) |
| CT18 | Typhi | Human | 1993 | [NC_003198.1](http://www.ncbi.nlm.nih.gov/nuccore/16758993) |
| LT2 | Typhimurium | Human | 1940s | [NC_003197.1](http://www.ncbi.nlm.nih.gov/nuccore/16763390) |
| SA20092095 | Wandsworth | Shrimp | 2009 | [NZ_CP019417.1](https://www.ncbi.nlm.nih.gov/nuccore/NZ_CP019417.1) |
| 2511STDY5462413 | Weltevreden | Human stool | 2010 | [NZ_LN890520.1](https://www.ncbi.nlm.nih.gov/nuccore/NZ_LN890520.1) |
| 4,[5],12:i:- | 4,[5],12:i:- | Roaster Swine | 2015 | [NZ_CP040686.1](https://www.ncbi.nlm.nih.gov/nuccore/NZ_CP040686.1) |
| *Salmonella* subsp.salamae |  |  |  |  |
| 1009S1 | 4,5,12:a:- | Human stool | 2012 |  |
| 2073S1 | 4,5,12:a:- | Human stool | 2012 | SAL_QB3388AA_AS |
| 2107S1 | 4,5,12:a:- | Human stool | 2012 | SAL_QB3389AA_AS |
| NCTC9930 | 40:z4,z24:z39 |  | 1885/1956 | [NZ_LS483456.1](https://www.ncbi.nlm.nih.gov/nuccore/NZ_LS483456.1) |
| RSE09 | 42:r:- | Ovis aries | 2018 | [NZ_CP034717.1](https://www.ncbi.nlm.nih.gov/nuccore/NZ_CP034717.1) |
| RSE36 | 42:r:- | Meat retail | 2018 | [NZ_CP034702.1](https://www.ncbi.nlm.nih.gov/nuccore/NZ_CP034702.1) |
| RSE42 | 42:r:- | Meat retail | 2018 | [NZ_CP034697.1](https://www.ncbi.nlm.nih.gov/nuccore/NZ_CP034697.1) |
| 1315K | 55:k:z39 | Chamaeleonidae | 1961 | [NZ_CP022139.1](https://www.ncbi.nlm.nih.gov/nuccore/NZ_CP022139.1) |
| SA20053897 | 56:b:1,5 |  |  | [NZ_CP029995.1](https://www.ncbi.nlm.nih.gov/nuccore/NZ_CP029995.1) |
| ST114 | 57:z29:z42 |  | 1970 | [NZ_CP022467.1](https://www.ncbi.nlm.nih.gov/nuccore/NZ_CP022467.1) |
| NCTC10310 | 58:1,z13,z28:1,5 | Human urine | 1884/1962 | [NZ_LS483477.1](https://www.ncbi.nlm.nih.gov/nuccore/NZ_LS483477.1) |
| FSW0196 | Fulica | Frozen silver fish | 2011 | SAL_BA2595AA_AS |
| DMA-1 |  | Mouse stool | 2010-2012 | [ATFA00000000.1](http://www.ncbi.nlm.nih.gov/nuccore/519151316) |
| NCTC5773 |  |  | 1800/2017 | [NZ_LR134141.1](https://www.ncbi.nlm.nih.gov/nuccore/NZ_LR134141.1) |
| NCTC8273 |  |  | 1800/2017 | [NZ_LR134154.1](https://www.ncbi.nlm.nih.gov/nuccore/NZ_LR134154.1) |
| NCTC9936 |  | 50 : z : e, n, x | 1885/1956 | [NZ_LS483475.1](https://www.ncbi.nlm.nih.gov/nuccore/NZ_LS483475.1) |
| *Salmonella* subsp.arizonae |  |  |  |  |
| NCTC7300 | 1,4:1,2,5 | Liver of poultry | 1800/1947 | [NZ_LR133910.1](https://www.ncbi.nlm.nih.gov/nuccore/NZ_LR133910.1) |
| NCTC7307 | 6:1,2,5 | Lung of poultry | 1800/1947 | [LS483466.1](https://www.ncbi.nlm.nih.gov/nuccore/LS483466.1) |
| RKS2983 | 62:z36:- |  | 1985 | [NZ_CP006693.1](https://www.ncbi.nlm.nih.gov/nuccore/NZ_CP006693.1) |
| RSK2980 | 62:z4,z23:- | Cornsnake | 1986 | [NC_010067.1](https://www.ncbi.nlm.nih.gov/nuccore/NC_010067.1) |
| SA19981204 | 63:g,z51:- |  |  | [NZ_CP029991.1](https://www.ncbi.nlm.nih.gov/nuccore/NZ_CP029991.1) |
| SA20100345 | O53 |  | 1995 | [NZ_CP022504.1](https://www.ncbi.nlm.nih.gov/nuccore/NZ_CP022504.1) |
| NCTC10047 |  |  | 1800/2017 | [NZ_LR134156.1](https://www.ncbi.nlm.nih.gov/nuccore/NZ_LR134156.1) |
| NCTC7306 |  |  | 1800/2017 | [NZ_LR134150.1](https://www.ncbi.nlm.nih.gov/nuccore/NZ_LR134150.1) |
| *Salmonella* subsp.diarizonae |  |  |  |  |
| SA20121591 | 48:i:z |  |  | [NZ_CP029989.1](https://www.ncbi.nlm.nih.gov/nuccore/NZ_CP029989.1) |
| MZ0080 | 50:k:z |  |  | [NZ_CP022142.1](https://www.ncbi.nlm.nih.gov/nuccore/NZ_CP022142.1) |
| 11-01853 | 60:r:z | Human | 2010 | [NZ_CP011289.1](https://www.ncbi.nlm.nih.gov/nuccore/NZ_CP011289.1) |
| 11-01854 | 60:r:z | Human alloplastic vascular graft | 2011 | [NZ_CP011292.1](https://www.ncbi.nlm.nih.gov/nuccore/NZ_CP011292.1) |
| 11-01855 | 60:r:z | Humanstool | 2011 | [NZ_CP011288.1](https://www.ncbi.nlm.nih.gov/nuccore/NZ_CP011288.1) |
| NCTC10381 | 61:i:z |  | 1884/1962 | [NZ_LS483474.1](https://www.ncbi.nlm.nih.gov/nuccore/NZ_LS483474.1) |
| SA20044251 | 65:c:z |  | 1993 | [NZ_CP022135.1](https://www.ncbi.nlm.nih.gov/nuccore/NZ_CP022135.1) |
| HZS154 |  | Human stool | 2016 | [NZ_CP023345.1](https://www.ncbi.nlm.nih.gov/nuccore/NZ_CP023345.1) |
| *Salmonella* subsp.houtenae |  |  |  |  |
| RKS3027 | 16:z4,z32:-- | Human blood | 1986 | [NZ_ANHR00000000.1](http://www.ncbi.nlm.nih.gov/nuccore/489044914) |
| NCTC10401 | 43:z4, z23:- |  |  | [NZ_LS483478.1](https://www.ncbi.nlm.nih.gov/nuccore/NZ_LS483478.1) |
| 01-0133 |  |  | 2001 | [NZ_AOXJ00000000.1](http://www.ncbi.nlm.nih.gov/nuccore/554691205) |
| ATCC BAA-1581 |  | Human | 2005 | [NZ_AGRM00000000.1](http://www.ncbi.nlm.nih.gov/nuccore/423138530) |
| NCTC7318 |  |  | 1800/2017 | [NZ_LR134159.1](https://www.ncbi.nlm.nih.gov/nuccore/NZ_LR134159.1) |
| *Salmonella* subsp.indica |  |  |  |  |
| 396-83 | 1,6,14:a:e,n,x |  | 1983 | [GCA_002066855.1](https://www.ncbi.nlm.nih.gov/assembly/GCA_002066855.1) |
| BCW_1559 | 11:b:1,7 |  |  | [GCA_002035225.1](https://www.ncbi.nlm.nih.gov/assembly/GCA_002035225.1) |
| 347-78 | 11:b:e,n,x |  | 1978 | [GCA_002066875.1](https://www.ncbi.nlm.nih.gov/assembly/GCA_002066875.1) |
| 1121 | 6,14,25:z10:1,[2],7 |  |  | [NZ_AOXI00000000.1](http://www.ncbi.nlm.nih.gov/nuccore/554685972) |
| BCW_1558 | 6,14,25:z10:1,[2],7 |  |  | [GCA_002035485.1](https://www.ncbi.nlm.nih.gov/assembly/GCA_002035485.1) |
| CFSAN059887 |  | Cattle stool | 2012 | [GCA_002266165.1](https://www.ncbi.nlm.nih.gov/assembly/GCA_002266165.1) |
| NCTC12420 |  |  | 1800/2017 | [GCA_900456865.1](https://www.ncbi.nlm.nih.gov/assembly/GCA_900456865.1) |
| *Salmonella bongori* |  |  |  |  |
| RKS3044 | 48:z41:- | Parakeet | 1976 | [CP006692.1](http://www.ncbi.nlm.nih.gov/nuccore/657146798) |
| N268-08 |  | Human | 2008 | [NC_021870.1](http://www.ncbi.nlm.nih.gov/nuccore/526225953) |
| NCTC 12419 |  | Frog | 1972 | [NC_015761.1](http://www.ncbi.nlm.nih.gov/nuccore/339998036) |

**Supplementary Table 4.** Mutations of *mdcABCDE* in 1009S1 and gene complementation results

| Gene*(bps) | SNPs | Amino acid mutations | Malonate metabolism in gene complementation strain |
| --- | --- | --- | --- |
| *mdcA* (1656) | 140 | 14 | negative |
| *mdcB* (858) | 105 | 18 | negative |
| *mdcC* (219) | 17 | 4 | negative |
| *mdcD* (834) | 63 | 4 | negative |
| *mdcE* (804) | 93 | 17 | negative |
| *mdcAB* | - | - | negative |
| *mdcCDE* | - | - | positive |

*:The genome of subspecies *arizonae* 62:z4,z23:- as reference

**Supplementary Table 5** *Salmonella* new serovar was cytotoxic for RAW264.7 macrophage cell line

| Strain | OD_577_* | *P* value** |
| --- | --- | --- |
| 1009S1 | 0.230±0.03 |  |
| 1009S1△SPI-1 | 0.475±0.05 | *p*＜0.05 |
| SL1344 | 0.216±0.02 | *p*＞0.05 |
| DH5α | 0.527±0.06 | *p*＜0.01 |
| Uninfected | 0.537±0.08 |  |

* OD_577_ values ± standard deviation from a representative 96-well tissue culture plate assay at 20 h postinfection. The experiment was repeated three times.

** A Student *t* test was used to determine the significance of the differences between *Salmonella* new serovar 1009S1 and isogenic SPI-1 mutant strain, 1009S1 and SL1344 strain, 1009S1 and DH5α strain, respectively.

**Supplementary Table 6**. Primers and strains used in gene deletion and complementation in the study

| Gene* and strain | Primers(5’-3’) |
| --- | --- |
| *mdcAB* | CATGCCTGCAGATGTTATCTGGGCAAACGC  GCTTGCGGCCGCCGATTAAGACGCGGCTGGC |
| *mdcCDE* | CATGCCTGCAGATGGAGGTGTTATTCACCGC  GCTTGCGGCCGCTTACCAGTTCGCCCGCAT |
| *mdcABCDE* | CATGCCTGCAGATGTTATCTGGGCAAACGC  GCTTGCGGCCGCTTACCAGTTCGCCCGCAT |
| hilC_F | CGCACTCGAGGCCTCATATAAACGAGTGCC |
| orgA_R | ACAGCCGATCAGTAAAAAGACTCGCCTGTTGAGGGG |
| invH_F | CCCCTCAACAGGCGAGTCTTTTTACTGATCGGCTGT |
| invH_R | GCGGTGGCGGCCGCGGGCTTTCAGCTTGAGATACT |
| espB_F | CGCACTCGAGTTGCCCCATACGATTCTGAAC |
| espB_R-tet | GATAAGCTGTCAAACATGAGAAATGCTGAACGCTATCGATACA |
| tetF-espB | GTATCGATAGCGTTCAGCATTTCTCATGTTTGACAGCTTATC |
| tetR-espH | ATCAGAATACGATGTTACCATCAAGGGTTGGTTTGCGCATT |
| espH_F-tet | AATGCGCAAACCAACCCTTGATGGTAACATCGTATTCTGATATG |
| espH_R | GCGGTGGCGGCCGCTTACAGATGATGTATAAAATTCGG |
| aec16_F | CGCACTCGAGGAACTTGTTTCCACCCGCACT |
| aec16_R-cat | TAGCAGAGCGAGGTATGTAGACGGCGATCGGTGATCAGTAC |
| catF- aec16 | GTACTGATCACCGATCGCCGTCTACATACCTCGCTCTGCTAA |
| catR- aec30 | GCTTTTCGGTTGTGGTTCAAGCCCTCATCAGTGCCAACATA |
| aec30_F-cat | TATGTTGGCACTGATGAGGGCTTGAACCACAACCGAAAAGC |
| aec30_R | GCGGTGGCGGCCGCTTTCCCGGTTTTTATGTCCAC |
| 1009S1 | Wide-type strain, *S.* 4,5,12:a:- |
| 1009S1ΔSPI-1 | The region from *hilC* to *invH* was deleted in 1009S1 strain |
| 1009ΔLEE | The region including TIR, LEE1, LEE2, LEE3 and half of LEE4, was deleted and inserted with a *tet* gene in 1009S1 strain |
| 1009S1ΔSPI-1ΔLEE | The region from *hilC* to *invH* was deleted in 1009ΔLEE |
| 1009ΔACE | The region from *aec16* to *ace30* genes was deleted and inserted with a *cat* gene in 1009S1 strain |
| 1009S1::mdcABCDE | 1009S1 strain was complemented with *mdcABCDE* genes |
| 1009S1::mdcAB | 1009S1 strain was complemented with *mdcAB* genes |
| 1009S1::mdcCDE | 1009S1 strain was complemented with *mdcCDE* genes |

*:The genome of subspecies *arizonae* 62:z4,z23:- as reference for amplification of *mdcABCDE*.


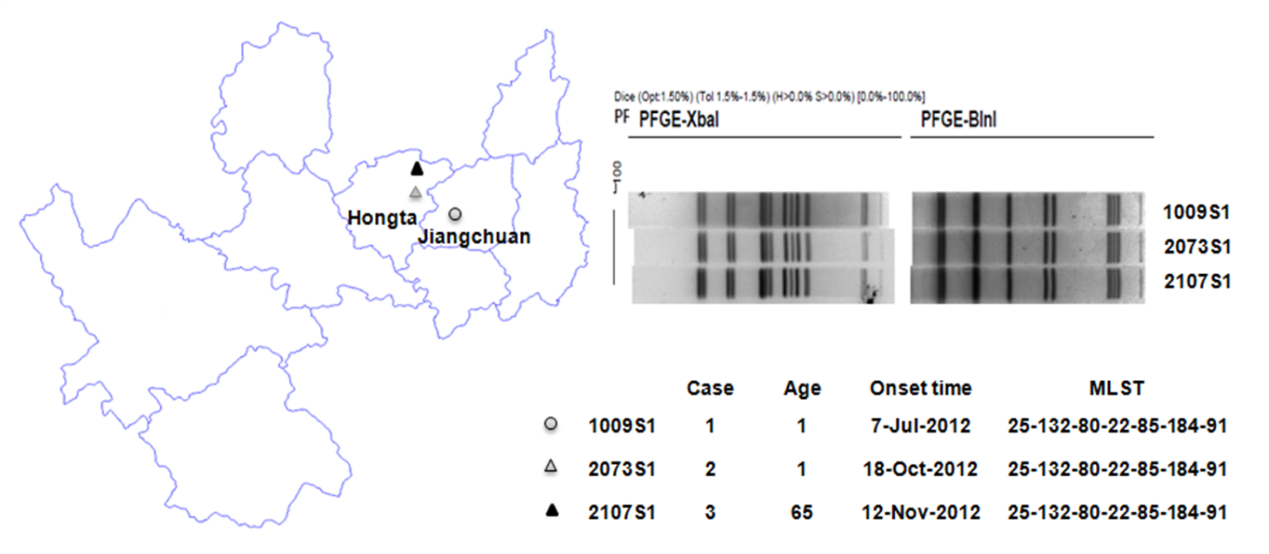


**Supplementary Figure 1.** Epidemiological and molecular characteristics of the three cases and *S.* 4,5,12:a:- strains in Yuxi, Yunnan. The map shows the geographical location of the three cases and the identical PFGE patterns of the strains digested with two endonucleases, *Xba*I and *Bln*I. The epidemiological data and MLST types of these cases are listed at the bottom of the figure.


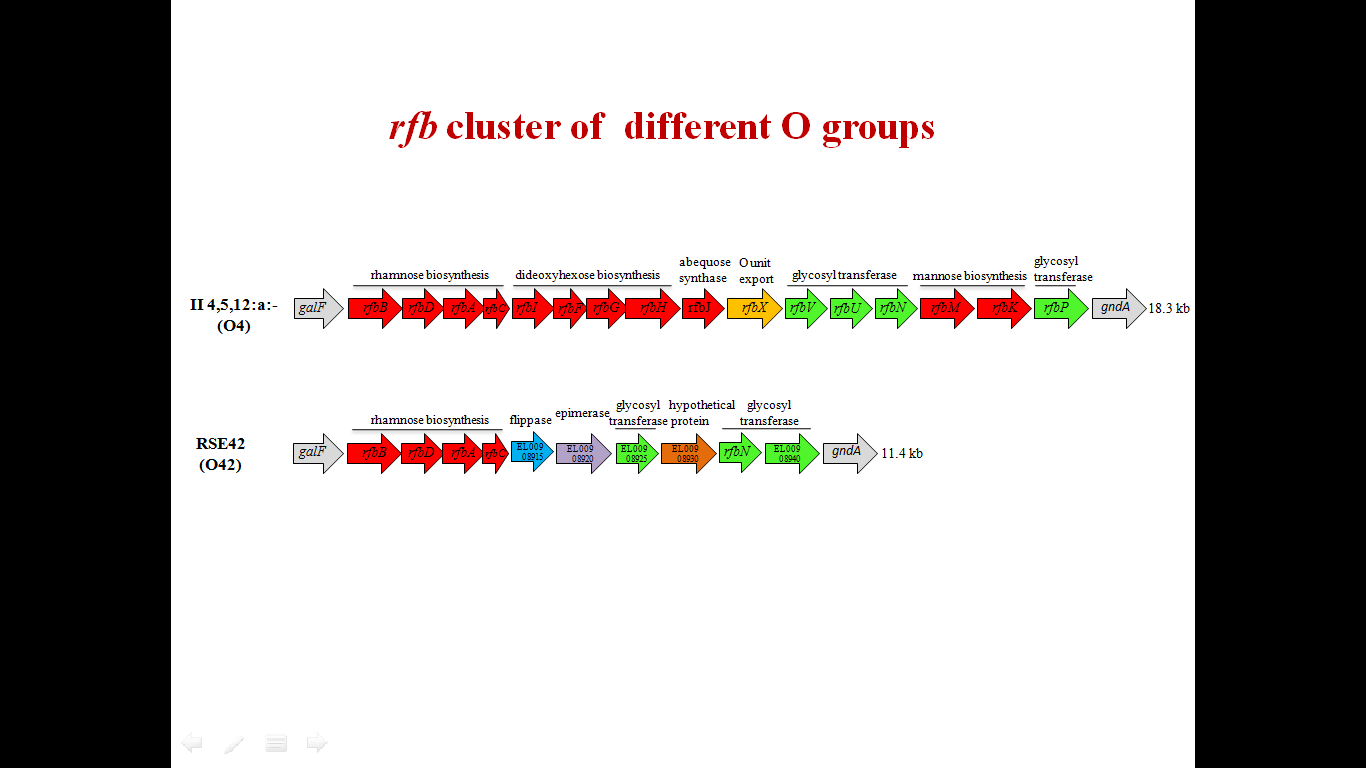


**Supplementary Figure 2.** The structure of the two *rfb* gene clusters in *Salmonella*

The O groups O4 (II 4,5,12:a) and O42 (RSE42, belonging to subspecies II), are encoded by *rfb* genes composed of different gene clusters. The *rfb* gene cluster was located between the *galF* and *gndA* genes, and was subtracted from the genome sequence and annotated basing on the blast results, which showing nearly 100% identity with the O encoding sequence from Agona strain SG17-135 (whose O antigen is 4,5,12). Different colored ORFs represent different functions of gene products: red represents hexose synthesis, and green represents glycosyltransferase.


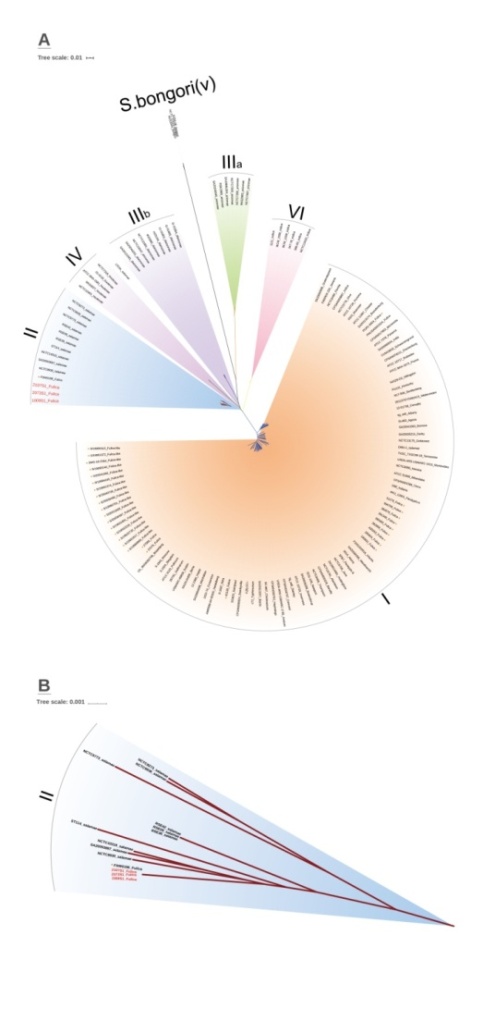


**Supplementary Figure 3.** Phylogenetic tree of 144 genomes of represented *Salmonella* strains with different subspecies and serotypes, including three *S.* 4,5,12:a:- strains

A: A maximum likelihood tree was constructed according to the SNPs identified among 144 genomes of *Salmonella* strains, including the three *S.* 4,5,12:a:- strains sequenced in this study. B: A scale up of clade II. The three *S.* 4,5,12:a:- strains (red) clustered together to form a separate branch (red, 100 bootstrap replicates, 3B).With the exception of some strains, all isolates were clustered into seven groups (I, II, IIIa, IIIb, IV, V, VI), which is concordant with their traditional taxonomy (3A).The genome codes are presented as strain code_serovar or subspecies. The strains with the same antigenic formula of *S.* 4,5,12:a:-, including Fulica and Fulica-like strains, are marked with orange dots. The dendrogram was constructed with iTOL (<https://itol.embl.de>).


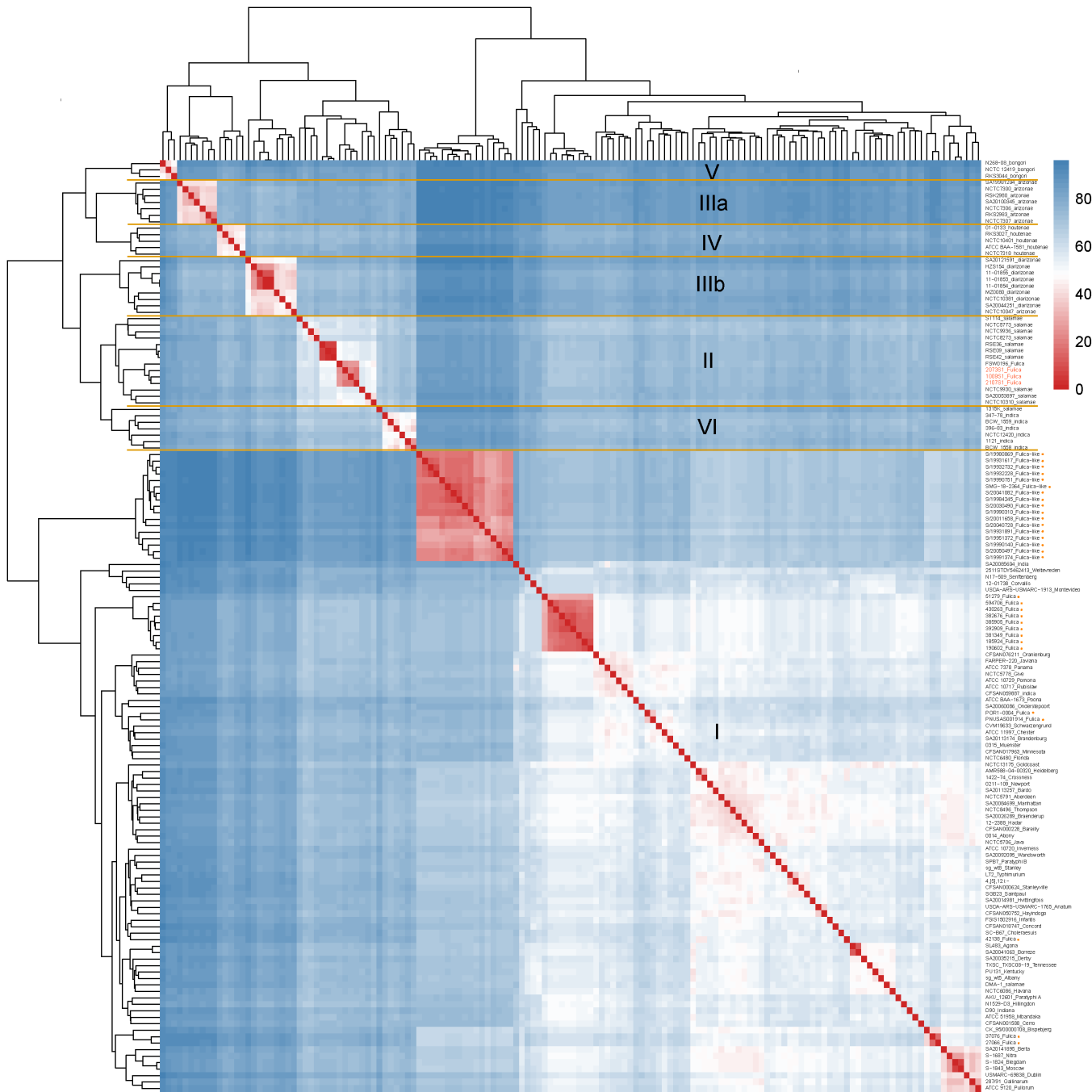


**Supplementary Figure 4.** Pangenome dendrogram of 144 *Salmonella* strains

A heatmap was constructed based on the pangenome of the 144 strains. With the exception of some strains, such as 2073S1, 1009S1, 2107S1, FSW0196, and DMA-1, all isolates were clustered into seven groups (I, II, IIIa, IIIb, IV, V, VI), which is concordant with their traditional taxonomy. The three new 4,5,12:a:- strains (2073S1, 1009S1and 2107S1) are labeled in red, and the same-antigenic formula strains, Fulica and Fulica-like, are marked with orange dots. The color bar shows genome distance; red indicates a close distance, and blue indicates a long distance.


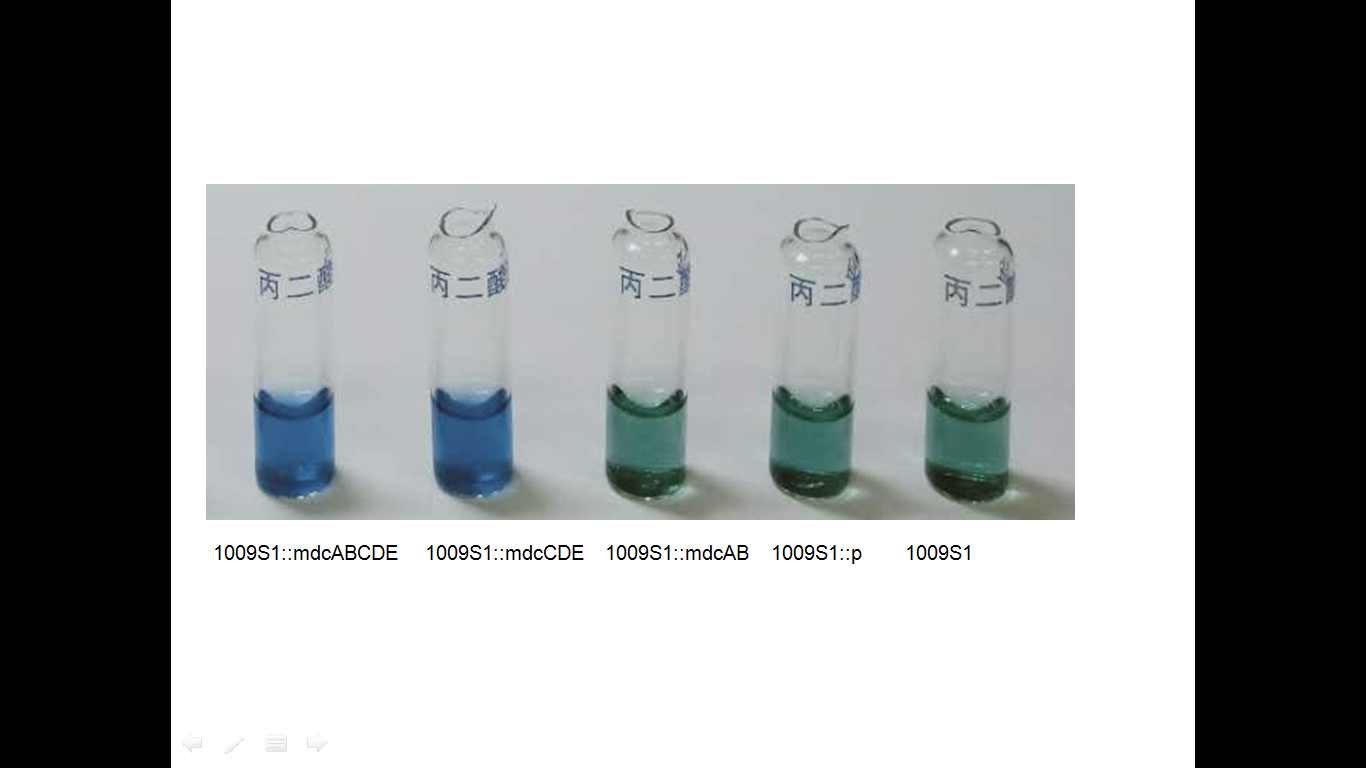


**Supplementary Figure 5.** Gene complementation of the malonate metabolism system

Different standard genes of *mdcABCDE* were cloned into 1009S1 to complement corresponding mutation genes, and the resulting complement strains were subjected to malonate metabolism tests. 1009S1::mdcABCDE comprised1009S1 complemented with *mdcABCDE* genes. 1009S1::mdcCDE comprised1009S1 complemented with *mdcCDE* genes. 1009S1::mdcAB comprised 1009S1 complemented with *mdcAB* genes. 1009S1::p comprised1009S1 containing the plasmid. The green color indicates a negative reaction, and the blue color indicates a positive reaction.


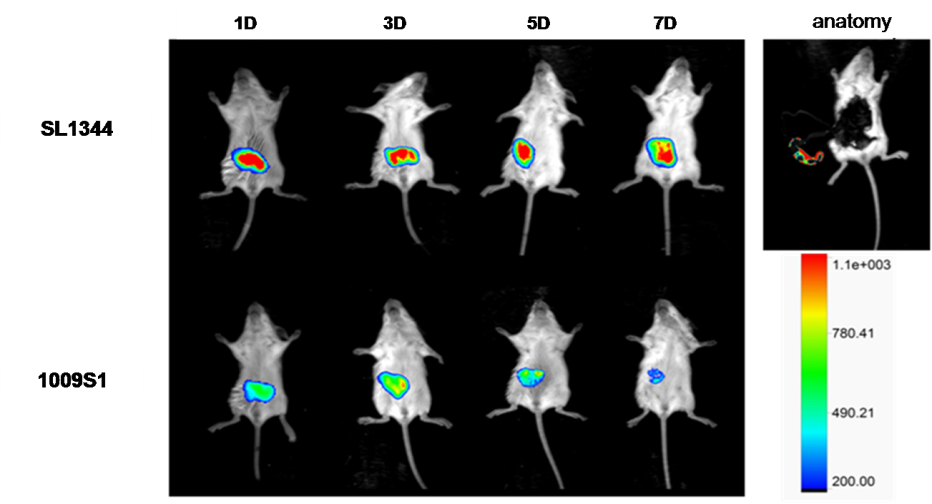


**Supplementary Figure 6.** Bioluminescence imaging of mice infected with *Salmonella* strains

The whole body of infected mice was scanned at 1, 3, 5 and 7 days after inoculation. In the SL1344-lux group, steady abdominal and intestinal signals were observed over seven days. Signals were also observed in strain 1009S1-lux-infected mice, but they were lower than those in SL1344-lux-infected mice and decreased over time. No signal was detected in the liver or spleen, which could be related to the weak transmission of luciferase in the experiments. The figure shows one representative mouse for each tested strain. The uninfected negative control mice showed no bioluminescence signal (data not shown). The color bar on the bottom right shows the intensity of the bioluminescence. The anatomy figure shows an example of the bioluminescence signals in the intestine after SL1344 infection.


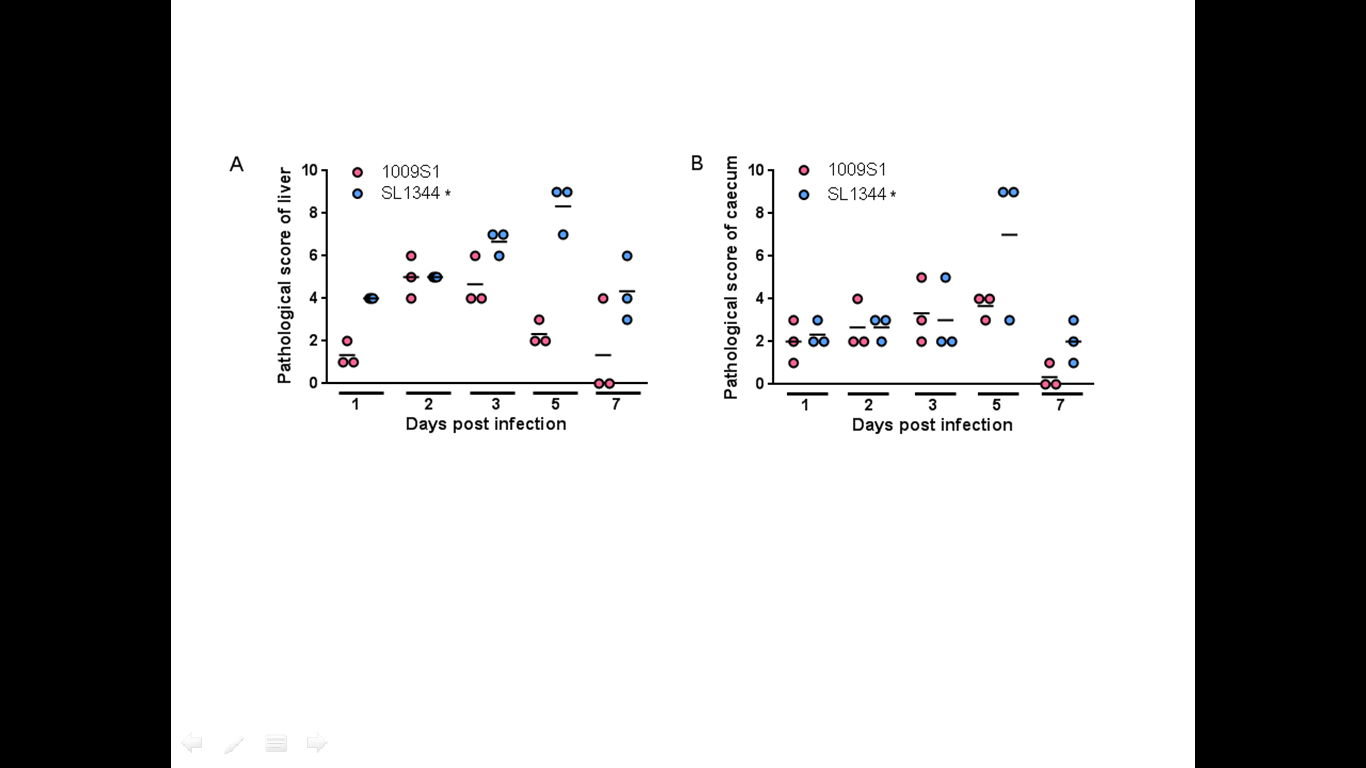


**Supplementary Figure 7.** Composite pathological score of liver and caecum (H&E)

All H&E (haematoxylin and eosin staining) sections were blindly evaluated by a veterinary pathologist using a pathological score system (see details in methods) and a composite sore of liver and caecum from different treatment groups was obtained. Figure A shows the composite sores of pathological change of liver from 1009S1 and SL1344 infected mice at days 1, 2, 3, 5 and 7 post infection (3 mice for each group). Figure B shows the composite sores of pathological change of caecum from 1009S1 and SL1344 infected mice at days 1, 2, 3, 5 and 7 postinfection (3 mice for each group). *: At the indicated time point the tissue sections were prepared from living mice except at day 5 postinfected with SL1344, at day 5 p.i. the tissue section was prepared from two dead mice and one living mouse.

**Supplementary Methods**

**Pangenome dendrogram construction**

A heatmap was constructed based on the pangenome to explore the genome compositions of 144 strains (Supplementary Table 2). First, we obtained a nonredundant homologous gene set from all selected strains by using cd-hit. Then, we constructed a matrix, with the rows including stains and the columns including nonredundant homologous genes. If a strain has the homologous gene, 1 was entered in the corresponding position in the matrix; otherwise, 0 was entered. The distance matrix was generated by computing the distance among strains based on the previous matrix. Finally, the heatmap library of the R programming language was used to draw the heatmap based on the distance matrix.

**Colonization assays in mice intestine using bioluminescence imaging**

The lux-labelled strain 1009S1-lux and SL1344-lux were constructed. The promoter region of the housekeeping gene *dnaK* was amplified from strain 1009S1 using a primer pair (5’CGGGGTACCCGGGTTTAATAAGCAATTACC3’and 5’CGCGGATCCCTAAACGTCTCCACTAAAAAT3’), digested with *Kpn*I and *BamH*I and cloned into pXEN-luxCDABE to generate the recombinant plasmid pXEN-dnaK-luxCDABE, which was subsequently transformed into strains 1009S1 and SL1344. Colonies of 1009S1 and SL1344 with high bioluminescence levels were verified by sequencing, and designated as 1009S1-lux and SL1344-lux, respectively. The growth of both strains was detected in either LB or LPM pH 5.5 culture medium containing ampicillin (100 µg/ml) to ensure no significant growth difference between the strains. Both strains were orally administered to the mice at a dose of 1.8×10^7^ CFUs. At postinfection days 1, 3, 5 and 7, the mice were imaged for bioluminescence with an *in vivo* FX PRO imaging system (Bruker, USA) after anesthesia. Three male BALB/c mice were included in each strain group.

**Gene complementation of the malonate metabolism system**

To ensure the target gene expression in 1009S1, we used the recombinant plasmid pXEN-dnaK-luxCDABE as the original expression vector, different gene fragments from gene cluster *mdcABCDE* were amplified by corresponding primers (Supplementary Table 5) with Sa12165 (a subspecies *arizonae* strain positive for malonate metabolism) as the template, then were digested with and cloned into the expression vector, followed by transfer into 1009S1. The 10 resulting complemented strains (Supplementary Table 5) were subjected to malonate metabolism tests.

**Gene deletion of LEE locus, ACE T6SS and SPI-1 in *Salmonella* new serovar**

To delete the most of LEE locus in 1009S1, the upstream fragment, tetracycline gene and downstream fragment were amplified by the primer sets: espB_F/espB_R-tet, tetF-espB/tetR-espH and espH_F-tet/espH_R. The PCR products was digested with *Xho*I and *Not*I and then cloned into a suicide vector pMW91 (doi:10.1016/j.micpath.2015.12.004), the recombinant plasmid pMW-LEE was transformed into 1009S1 by conjugation experiment. The potential LEE in-frame deletion strain was selected on LB plates containing 10% sucrose and 4 µg/ml tetracycline, the resulting LEE deletion strain 1009S1ΔLEE (nearly 30kb, the region covering entire TIR, LEE1, LEE2, LEE3 and most of LEE4 was deleted and replaced by a tetracycline resistance gene *tet*) was confirmed by PCR and gene sequencing. A similar procedure was performed to obtain the most of ACE genes deletion strain 1009S1ΔACE (approximately 28kb, the region from genes *aec16* to *ace30* containing the ATPase of T6SS clpV, was deleted and replaced by a chloramphenicol resistance gene *cat*), most of SPI-1 genes deletion strain 1009S1ΔSPI-1 (the region from genes *hilC* to *invH* was deleted) and double mutant strain 1009S1ΔSPI-1ΔLEE (most of SPI-1 genes were deleted in 1009S1ΔLEE strain). The above four isogenic strains were subjected to invasion assay to epithelial cells and competitive assay in mouse model. The primers and strains for gene deletion were listed in Supplementary Table 6. The primer sets of aec16_F/aec16_R-cat, catF-aec16/catR-aec30 and aec30_F-cat/aec30_R were used for ACE deletion, and the primer sets of hilC_F/orgA_R, invH_F/invH_R used for SPI-1 deletion. The concentration of chloramphenicol and ampicillin used in this study was 10 µg/ml and 100 µg/ml, respectively.
